# Changes in sputum microbiota during treatment for nontuberculous mycobacterial pulmonary disease

**DOI:** 10.1038/s41598-023-47230-5

**Published:** 2023-11-13

**Authors:** Bo-Guen Kim, Jin Young Yu, Su-Young Kim, Dae Hun Kim, Byung Woo Jhun

**Affiliations:** 1 Division of Pulmonary Medicine and Allergy, Department of Internal Medicine, Hanyang University College of Medicine, Seoul, Korea; 2 Department of Medicine, Samsung Medical Center, Sungkyunkwan University School of Medicine, Seoul, South Korea; 3 Division of Pulmonary and Critical Care Medicine, Department of Medicine, Samsung Medical Center, Sungkyunkwan University School of Medicine, 81 Irwon-Ro, Gangnam-Gu, Seoul, 06351 South Korea

**Keywords:** Clinical microbiology, Diseases, Medical research

## Abstract

Limited data exist on longitudinal changes in the sputum bacterial microbiome during treatment in nontuberculous mycobacterial pulmonary disease (NTM-PD) patients. We prospectively collected serial sputum samples from 14 NTM-PD patients during treatment, at the start (n = 14) and at 1 (n = 10), 3 (n = 10), 6 (n = 12), and 12 (n = 7) months. The bacterial microbiome changes were analyzed using 16S rRNA sequences (V3–V4 regions). Subgroup analysis included culture conversion (n = 9) and treatment refractory (n = 5) groups. In all patients, sputum alpha-diversity (ACE, Chao1, and Jackknife) significantly decreased during antibiotic treatment at 1, 3, 6, and 12 months compared to treatment initiation levels. Within the culture conversion group, genus/species-level beta-diversity showed differences at 1, 3, 6, and 12 months compared to treatment initiation (all *p* < 0.05). However, in the refractory group, there were no differences in beta-diversity at the genus/species levels in the sputum at any time point. In the linear discriminant analysis (LDA) effect sizes (LEfSe) analysis, the culture conversion group exhibited decreasing taxa at various levels (phylum/genus/species), but no significant increase in taxa was observed. LEfSe analysis of the refractory patient group revealed multiple taxa decreased during treatment. However, proportions of *Veillonella dispar* (LDA = 4.78), *Fusobacterium periodonticum* (LDA = 4.35), and *Pseudomonas aeruginosa* (LDA = 2.92) increased as the treatment period progressed in the refractory group. Sputum microbiota diversity decreases during NTM-PD treatment. In the culture conversion group, most taxa decrease, while some increase in the refractory group. These findings suggest that a distinct respiratory microbial community may exist in refractory NTM-PD patients compared to responsive antibiotic-treated patients.

## Introduction

Nontuberculous mycobacteria (NTM) are ubiquitous organisms found in natural environments and human communities^1^. NTM-pulmonary disease (PD) is a chronic respiratory infection caused by various NTM species, and its burden is increasing globally^2,3^. Among the more than 200 species of NTM, the *Mycobacterium avium* complex, primarily composed of *M. avium* and *M. intracellulare*, is the most common pathogen, followed by *M. abscessus,* in many countries^4–6^.

For the treatment of NTM-PD, long-term combination therapy with macrolide-based antibiotics is used, and patients with NTM-PD show heterogeneous responses^4,7,8^. While there are patients who easily achieve negative culture conversion after antibiotics, there are also patients whose disease is refractory, defined as persistent NTM culture positivity even after several months of antibiotic treatment^9–11^. Several studies have been conducted to elucidate the various treatment responses by investigating the clinical, immunological, and metabolic characteristics associated with treatment response. However, these phenomena have not been clearly explained thus far.

Recent studies have reported on the investigation of changes in the lung microbiome to elucidate the pathogenesis or exacerbation of respiratory diseases^12,13^. The microbiome, comprising microbial communities (e.g., bacteria, viruses, and fungi), has been reported to evaluate respiratory disease progression and pathogenesis^14^. Culture-independent techniques, such as targeted sequencing of the 16S ribosomal RNA (rRNA) gene, aid in identifying microbes, and in particular, 16S rRNA sequencing can be applied on a large scale for characterizing the various taxa present in a microbiome. Considering that the lung is not a sterile space and that NTM, as ubiquitous organisms, cause lung disease by interacting with the host's immune system, there is an increasing interest in investigating changes in the microbial environment in NTM-PD.

Although some microbiome studies using sputum, alveolar lavage fluid, or lung tissue from patients with NTM-PD have been published, these studies did not include a comparative analysis of the longitudinal microbiota according to the course of treatment^15–18^. Therefore, in this study, we investigated how the bacterial microbiota of sputum changes during antibiotic treatment in patients with NTM-PD. Additionally, we aimed to determine the differences in the microbial environment of sputum between patients who achieved culture conversion and those who remained refractory after treatment. Our findings can contribute to the characterization of microbial environments in the respiratory tract of patients with NTM-PD, providing insights into the impact of treatment on the microbiota.

## Results

### Study participants

The baseline characteristics of 14 NTM-PD patients are presented in Table 1. The majority of the patients (93%) were females and non-smokers. Half of the patients had *M. intracellulare* infection, while two patients had *M. avium* and two patients had *M. massiliense* infections. Three patients were co-infected with two NTM organisms. All patients exhibited the nodular bronchiectatic form of NTM-PD, and 57% of them had cavities. During antibiotic treatment, 9 patients (64%) achieved negative culture conversion, defined as three consecutive negative cultures at 4-week intervals, while 5 patients remained refractory with persistent NTM (online supplemental Table S1). Details of the antibiotics used in study patients are presented in online supplemental Table S2.

**Table 1:** Characteristics of 14 study patients at the time of starting antibiotics (n = 14).

Characteristics	Value
Age, years	58 (50–66)
Female	13 (93)
Body mass index, kg/m^2^	21.6 (19.7–23.0)
Never smoker	13 (93)
Etiology	
*M*. *intracellulare*	7 (50)
*M*. *avium*	2 (14)
*M. massiliense*	2 (14)
Coinfection^a^	3 (21)
Positive acid-fast bacilli smear	3 (21)
Radiological form of NTM-PD	
Nodular bronchiectatic form	14 (100)
Without cavity	6/14
With cavity	8/14
Treatment outcomes	
Culture conversion group^b^	9 (64)
Refractory group	5 (36)

Data are presented as number (percentage), or median (interquartile range).

NTM, nontuberculous mycobacteria; PD, pulmonary disease.

^a^*M. intracellulare* and *M. abscessus* (n = 1), *M. avium* and *M. abscessus* (n = 1), and *M. avium* and *M. intracellulare* (n = 1). ^b^Culture conversion within 12 months after antibiotic therapy.

When comparing the diversity between eight patients with cavities and six patients without cavities, neither alpha-diversity nor beta-diversity showed significant differences. We additionally compared the microbiomes between four patients aged 65 or older and ten relatively younger patients and found no significant differences in alpha- and beta-diversity between the two groups.

### Changes in taxa proportions, diversity, and specific taxa in all patients

We evaluated the changes in the serial sputum bacterial microbiome during the course of treatment in all patients. In terms of phylum, *Proteobacteria*, *Firmicutes*, and *Bacteroidetes* had the highest overall proportions. During treatment, the phyla *Saccaribacteria*, *Fusobacteria*, *Actinobacteria*, and *Spirochaetes* showed a tendency to decrease (online supplemental Fig. S1). At the genus level, several taxa decreased during treatment (online supplemental Fig. S2), including *Tannerella*, *Fusobacterium*, *Actinomyces*, *Porphyromonas*, *Saccharimonas*, and *Alloprevotella*. The proportion of *Pseudomonas* showed an increasing trend at 6 months and 12 months compared to the treatment start. The Lachnospiraceae family, an important gut taxon, decreased with antibiotic treatment.

**Figure 1:**
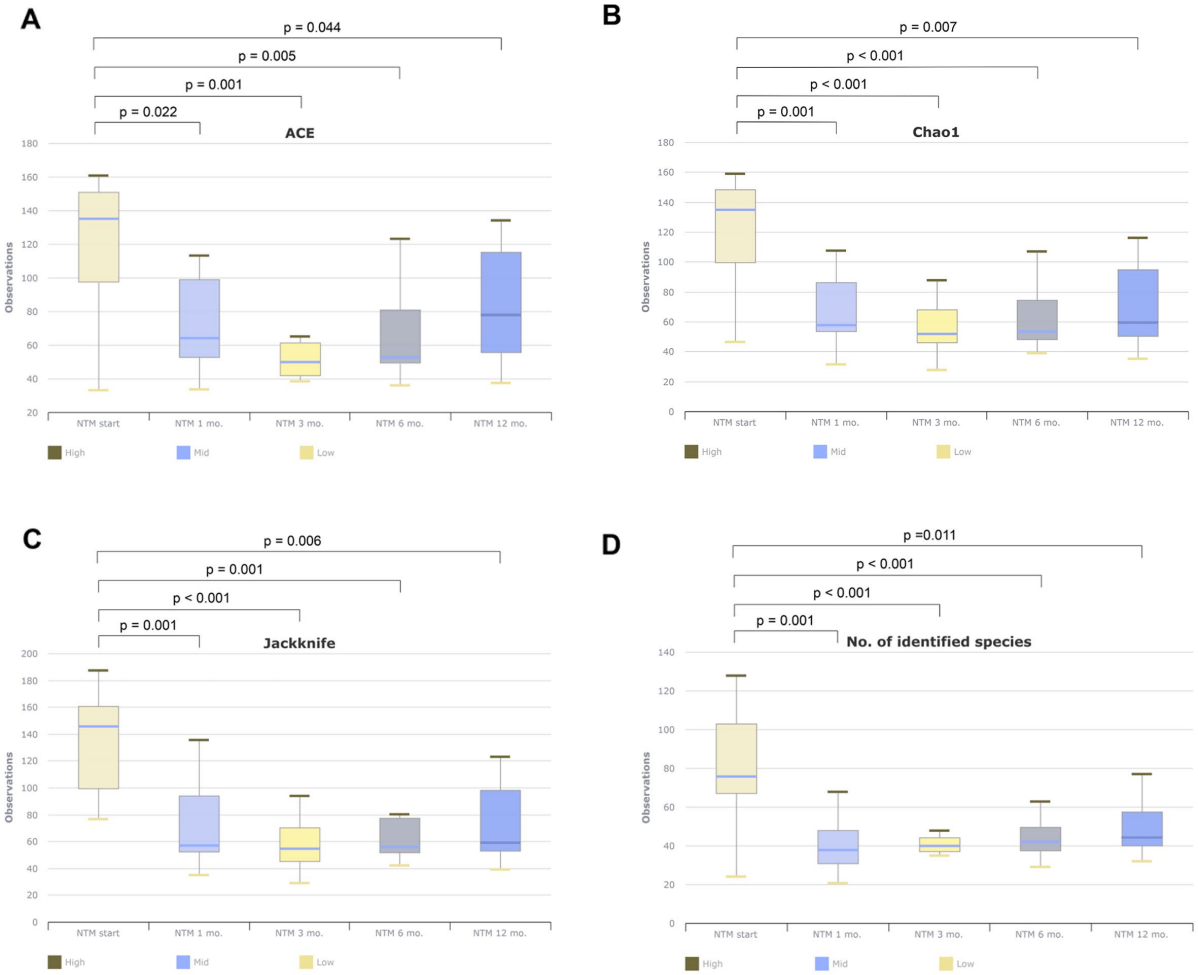
Change of alpha-diversity during antibiotics treatment in all patients.

During antibiotic treatment, alpha-diversity (ACE, Chao1, Jackknife, operational taxonomic unit [OUT]) showed a significant decrease in sputum at 1, 3, 6, and 12 months after treatment compared to the time of treatment (Fig. 1). In particular, alpha-diversity decreased significantly between 1 and 3 months after treatment initiation and reached a relatively stable plateau at 6 and 12 months.

In the genus-level beta-diversity analysis using Generalized UniFrac, compared to the treatment initiation, there was a significant distributional difference at 1 (*p* = 0.012), 3 (*p* = 0.018), and 6 months (*p* = 0.024). Also, at the species level, compared to the treatment initiation, there was a significant distributional difference at 1, 3, 6, and 12 months (all *p* < 0.05).

Linear discriminant analysis effect size (LEfSe) analysis was performed to identify the distinct microbial populations showing significant changes (Table 2). At the phylum, genus, and species levels, most of the taxa showed a tendency to decrease, while some taxa showed a tendency to increase in the distribution ratio. Specifically, *Veillonella dispar*, *Fusobacterium periodonticum*, *Streptococcus sinensis*, and *Pseudomonas aeruginosa* showed an increasing distribution at the species level.

**Table 2:** LEfSe analysis for bacterial taxa during antibiotic therapy in sputa samples from all 14 study patients.

Taxon name	Antibiotics start (%) (n = 14)	1 mo. (%) (n = 10)	3 mo. (%) (n = 10)	6 mo. (%) (n = 12)	12 mo. (%) (n = 7)	LDA effect size	*p*-value
Phylum							
*Fusobacteria*	5.11	1.47	4.42	4.08	4.69	4.28	0.044
*Spirochaetes*^b^	1.06	0.02	0.09	0.12	0.06	3.70	< 0.001^a^
Genus							
*Saccharimonas*^b^	5.10	0.26	0.17	2.48	1.47	4.38	< 0.001^a^
*Porphyromonas*^b^	4.94	0.53	2.70	1.06	1.70	4.28	0.027
*Alloprevotella*	1.08	0.02	0.89	0.78	1.26	3.92	0.049
*Aggregatibacter*^b^	0.59	0.00	0.00	0.90	0.04	3.69	0.001^a^
*Treponema*^b^	0.98	0.02	0.09	0.12	0.06	3.67	< 0.001^a^
*Dialister*^b^	0.35	0.01	0.03	0.16	0.13	3.24	0.001^a^
*Parvimonas*^b^	0.31	0.00	0.00	0.01	0.00	3.20	< 0.001^a^
Species							
*Veillonella dispar*	**5.89**	**13.01**	**11.98**	**8.48**	**6.89**	**4.49**	**0.044**
*Porphyromonas endodontalis*^b^	3.19	0.06	0.01	0.01	0.01	4.13	< 0.001^a^
*Fusobacterium periodonticum group*	**0.71**	**0.05**	**1.43**	**1.92**	**2.24**	**4.06**	**0.016**
*Fusobacterium nucleatum group*^b^	2.18	1.12	0.53	0.94	0.43	3.94	0.014
*Streptococcus sinensis group*	**0.70**	**2.36**	**1.97**	**1.88**	**2.13**	**3.90**	**0.031**
*Pseudomonas aeruginosa group*	**0.01**	**0.00**	**0.00**	**0.01**	**0.93**	**3.70**	**0.022**
*Aggregatibacter aphrophilus*^b^	0.34	0.00	0.00	0.90	0.04	3.69	0.006
*Rothia aeria*^b^	0.70	0.04	0.19	0.11	0.00	3.57	0.002
*Porphyromonas gingivalis*^b^	0.71	0.07	0.00	0.00	0.00	3.50	< 0.001^a^
*Haemophilus influenzae group*^b^	0.45	0.00	0.00	0.02	0.07	3.39	0.009
*Actinomyces graevenitzii*^b^	0.47	0.01	0.00	0.15	0.26	3.36	0.043
*Treponema medium group*^b^	0.34	0.00	0.04	0.02	0.01	3.25	0.003
*Neisseria elongata group*^b^	0.31	0.00	0.00	0.00	0.00	3.19	< 0.001^¶^
*Rothia dentocariosa*^b^	0.36	0.35	0.08	0.17	0.00	3.19	0.015
*Aggregatibacter segnis*^b^	0.24	0.00	0.00	0.00	0.00	3.11	0.019
*Treponema denticola*^b^	0.21	0.00	0.01	0.08	0.00	3.04	0.003
*Dialister pneumosintes*^b^	0.17	0.00	0.03	0.00	0.01	3.01	< 0.001^¶^
*Parvimonas micra*^b^	0.19	0.00	0.00	0.01	0.00	3.00	0.001^¶^

LEfSe, linear discriminant analysis effect size; LDA, linear discriminant analysis. LEfSe analysis included all taxa, including taxa with proportions < 1%. Taxa with an LDA effect size value > 3 were described. ^a^Adjusted *p*–value < 0.05; the Benjamini–Hochberg false discovery rate was applied to correct for multiple testing, and values of less than 0.05 were considered significant. ^b^This indicates that the proportions of the taxa decreased over the course of antibiotic treatment*.* Bold font indicates that the proportions of the taxa increased over the course of antibiotic treatment.

### Changes in taxa proportions, diversity, and specific taxa in the culture conversion group

**Figure 2:**
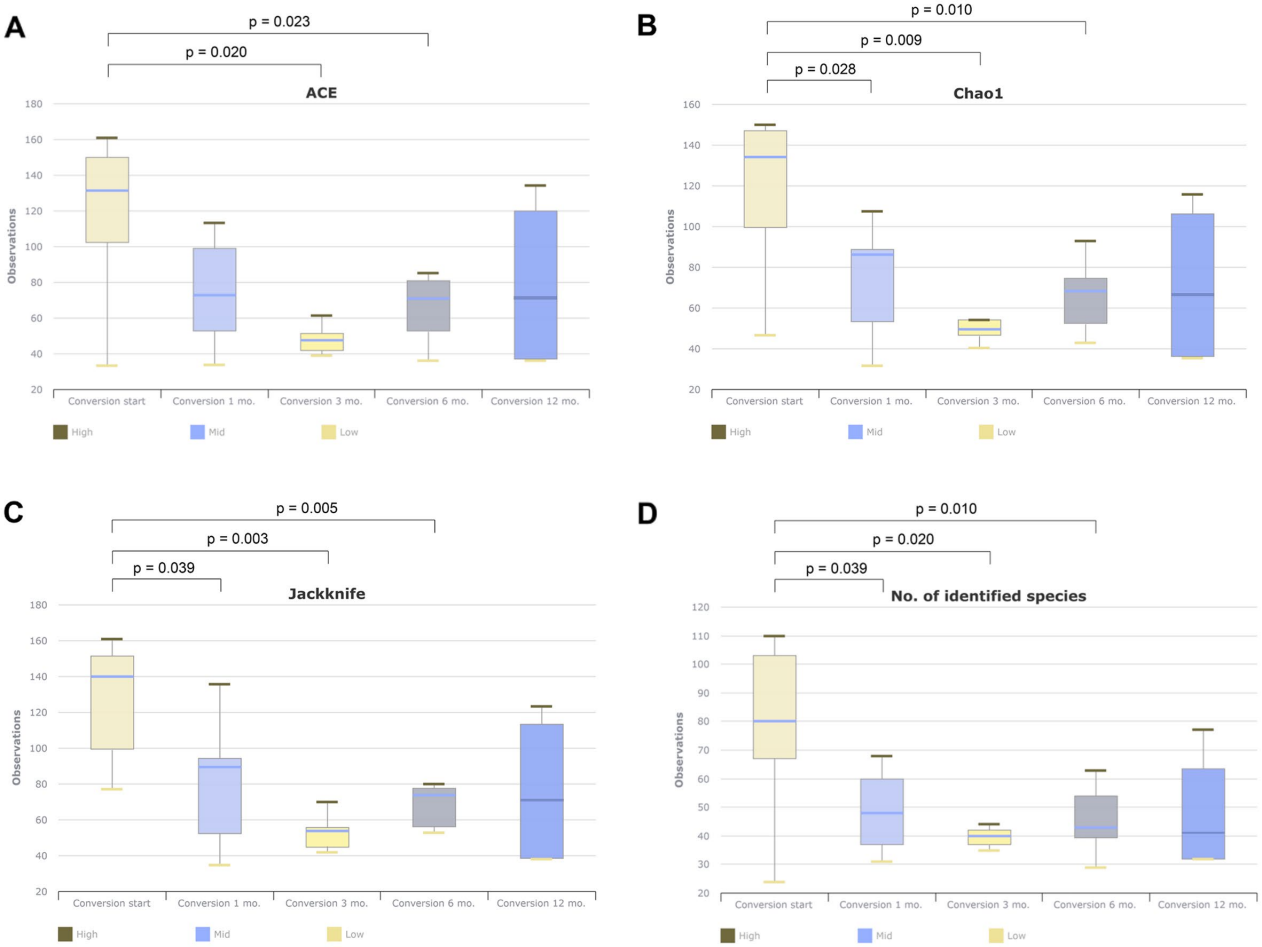
Change of alpha-diversity during antibiotics treatment in culture conversion group.

Microbiome analysis was performed based on the course of antibiotic treatment in the culture conversion group. Compared to results at the time of treatment initiation, alpha-diversity (ACE, Chao1, Jackknife, OTU) showed a significant decrease in sputum after 1, 3, and 6 months of treatment (Fig. 2). In the genus or species-level beta-diversity analysis, microorganism distribution in sputum showed significant differences at 1, 3, 6, and 12 months compared to treatment initiation (all *p* < 0.05).

LEfSe analysis was performed to identify the changing microbial distribution during treatment. Most taxa at the phylum, genus, and species levels showed a tendency to decrease, and no significant increase in bacterial taxa was observed (Table 3). A similar pattern was observed when comparing sputum samples from treatment initiation and the time of culture conversion, with taxa tending to decrease and no taxon showing an increase in sputum samples obtained on the day of culture conversion.

**Table 3:** LEfSe analysis for bacterial taxa according to treatment in sputa samples from 9 patients with culture conversion.

Taxon name	Antibiotics start (%) (n = 9)	1 mo. (%) (n = 5)	3 mo. (%) (n = 5)	6 mo. (%) (n = 7)	12 mo. (%) (n = 4)	LDA effect size	*p*-value
Phylum							
*Actinobacteria*^b^	5.83	4.48	1.48	5.49	1.23	4.35	0.027
*Spirochaetes*^b^	0.73	0.04	0.16	0.11	0.00	3.62	0.010
Genus							
*Rothia*^b^	3.37	1.56	0.44	3.60	0.25	4.20	0.021
*Alloprevotella*^b^	1.40	0.02	0.00	0.09	0.13	3.85	0.040
*Aggregatibacter*^b^	0.84	0.00	0.00	0.00	0.08	3.68	0.006
*Treponema*^b^	0.73	0.04	0.16	0.11	0.00	3.62	0.010
*Dialister*	0.27	0.00	0.04	0.06	0.25	3.20	0.026
*Parvimonas*^b^	0.23	0.00	0.00	0.00	0.00	3.09	0.002
*Moryella*^b^	0.11	0.00	0.00	0.27	0.05	3.09	0.017
Species							
*Porphyromonas endodontalis*^b^	3.80	0.12	0.00	0.00	0.00	4.32	0.026
*Rothia aeria*^b^	0.78	0.02	0.00	0.00	0.00	3.53	0.023
*Porphyromonas gingivalis*^b^	0.48	0.04	0.00	0.00	0.00	3.45	0.025
*Aggregatibacter aphrophilus*^b^	0.44	0.00	0.00	0.00	0.08	3.43	0.028
*Rothia dentocariosa*^b^	0.39	0.00	0.00	0.00	0.00	3.33	0.009
*Aggregatibacter segnis*^b^	0.38	0.00	0.00	0.00	0.00	3.31	0.035
*Neisseria elongata group*^b^	0.31	0.00	0.00	0.00	0.00	3.24	< 0.001^a^
*Dialister pneumosintes*^b^	0.18	0.00	0.04	0.00	0.20	3.04	0.011
*Stomatobaculum longum*^b^	0.11	0.00	0.00	0.23	0.05	3.02	0.017

LEfSe, linear discriminant analysis effect size; LDA, linear discriminant analysis. LEfSe analysis included all taxa, including taxa with proportions < 1%. Taxa with an LDA effect size value > 3 were described. ^a^Adjusted *p*–value < 0.05; the Benjamini–Hochberg false discovery rate was applied to correct for multiple testing, and values of less than 0.05 were considered significant. ^b^This indicates that the proportions of the taxa decreased over the course of antibiotic treatment.

### Changes in taxa proportions, diversity, and specific taxa in treatment refractory group

**Figure 3:**
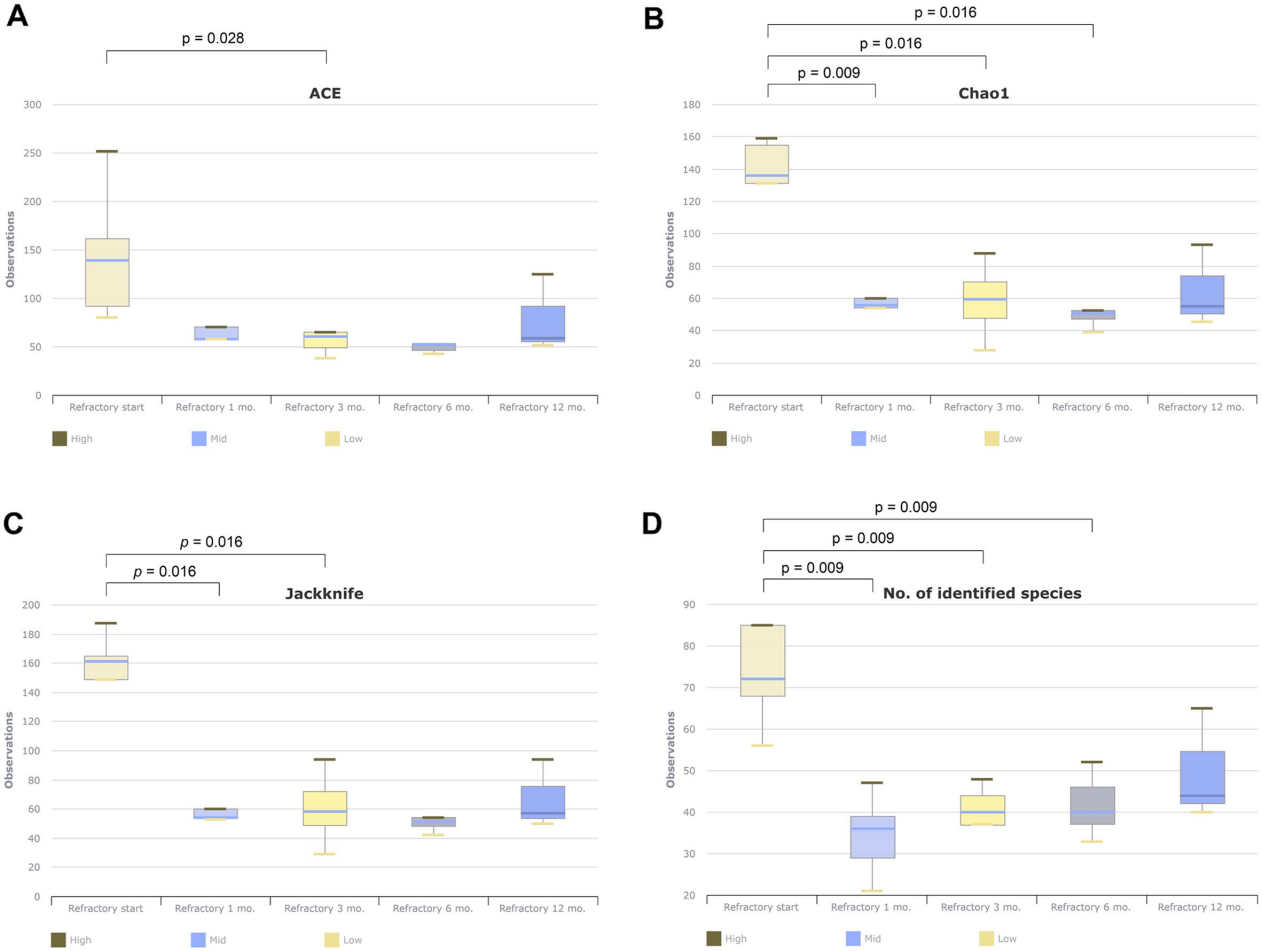
Change of alpha-diversity during antibiotics treatment in treatment refractory group.

Microbiome analysis was performed during antibiotic treatment in the refractory group. Compared to the time of treatment initiation, alpha-diversity (ACE, Chao1, Jackknife, OTU) tended to decrease in sputum after 1, 3, or 6 months of treatment (Fig. 3). However, there were no significant differences in beta-diversity at the genus or species levels at any time point in the sputum.

In the LEfSe analysis of the refractory patient group, proportions of multiple taxa were observed to decrease during the antibiotic treatment period. However, notably, three species showed significant increases in proportion over time (Table 4). The proportions of *Veillonella dispar* (linear discriminant analysis [LDA] effect size = 4.78), *Fusobacterium periodonticum* (LDA effect size = 4.35), and *Pseudomonas aeruginosa* (LDA effect size = 2.92) increased as the antibiotic treatment period elapsed in the refractory group (online supplemental Fig. S3). Furthermore, LEfSe analysis was conducted to compare the sputum samples of the refractory group at 6 months (n = 5) with the samples obtained on the day of culture conversion in the conversion group (n = 9). The analysis revealed a significant increase in the proportions of *Pseudomonas fulva* group species (LDA effect size = 4.60, *p* = 0.04, 6.16%) in the refractory patients' sputum at 6 months (*p* < 0.05).

**Table 4:** LEfSe analysis for bacterial taxa according to treatment in sputa samples from 5 refractory patients.

Taxon name	Antibiotics start (%) (n = 5)	1 mo. (%) (n = 5)	3 mo. (%) (n = 5)	6 mo. (%) (n = 5)	12 mo. (%) (n = 3)	LDA effect size	*p*-value
Phylum							
*Spirochaetes*^a^	1.64	0.00	0.02	0.12	0.13	3.90	0.005
Genus							
*Saccharimonas*^a^	7.22	0.00	0.00	0.00	2.27	4.60	0.006
*Campylobacter*^a^	1.66	0.18	0.28	1.60	0.43	3.88	0.015
*Treponema*^a^	1.42	0.00	0.02	0.12	0.13	3.84	0.007
Species							
*Veillonella dispar*	**1.88**	**13.98**	**10.46**	**9.16**	**9.07**	**4.78**	**0.018**
*Fusobacterium periodonticum group*	**0.84**	**0.02**	**2.18**	**3.70**	**4.03**	**4.35**	**0.044**
*Porphyromonas endodontalis*^a^	2.08	0.00	0.02	0.02	0.03	3.94	0.004
*Treponema medium group*^a^	0.58	0.00	0.00	0.00	0.03	3.50	0.007
*Treponema denticola*^a^	0.38	0.00	0.02	0.12	0.00	3.31	0.035
*Campylobacter gracilis*^a^	0.26	0.00	0.00	0.06	0.00	3.19	0.002
*Prevotella dentalis group*^a^	0.06	0.00	0.00	0.00	0.00	2.92	0.018
*Pseudomonas aeruginosa group*	**0.00**	**0.00**	**0.00**	**0.00**	**0.07**	**2.92**	**0.007**

LEfSe, linear discriminant analysis effect size; LDA, linear discriminant analysis. LEfSe analysis included all taxa, including taxa with proportions < 1%. Taxa with an LDA effect size value of around 3 or higher were described. ^a^This indicates that the proportions of the taxa decreased over the course of antibiotic treatment*.* Bold font indicates that the proportions of the taxa increased over the course of antibiotic treatment.

## Discussion

In our study, the bacterial microbiome was analyzed from sputum collected longitudinally from patients with NTM-PD during antibiotic treatment. As the antibiotic treatment progressed, a significant decrease in alpha-diversity was observed. Furthermore, unlike the culture-converted NTM-PD group, in which taxa in sputum showed a tendency to decrease during the course of antibiotic treatment, several taxa increased in the refractory NTM-PD group. These findings suggest that a more distinct microbial community may exist in the lower respiratory tract of patients with refractory NTM-PD compared to patients who respond well to antibiotic treatment.

In our study, in terms of microbiological diversity, alpha-diversity significantly decreased as antibiotic treatment progressed in all NTM-PD patients. Considering that the treatment of NTM-PD includes a macrolide-based multidrug therapy, the decrease in alpha-diversity after antibiotic use is expected. In previous studies, an association between a decrease in diversity and disease deterioration has been reported in other respiratory diseases. However, our findings suggest that relying solely on the decrease in alpha-diversity has limitations in predicting the treatment response of NTM-PD, considering the decreasing alpha-diversity observed in both the conversion group and the refractory group. Notably, in the refractory group, unlike the conversion group, there was no significant change in beta-diversity observed despite antibiotic treatment, possibly due to NTMs persisting in the refractory group. However, given the small sample size of the refractory group, it is challenging to definitively conclude that the lack of change in beta-diversity is a consistent phenomenon. If confirmed in a larger patient population, beta-diversity distance could potentially serve as a predictor of treatment response.

Notably, during the antibiotic treatment of the NTM-PD patients, species such as *Veillonella dispar*, *Fusobacterium periodonticum*, and *Pseudomonas aeruginosa* tended to increase in the refractory group. This phenomenon may result from dysbiosis due to prolonged antibiotic use without NTM eradication, or these species may serve as organisms that provide a favorable microbial environment for the survival of NTM. On the other hand, in the conversion group, most taxa decreased while none showed a significant increase during antibiotic treatment. This phenomenon may be due to the antibiotics for NTM-PD or the maintenance of a stable microbial environment by preventing the proliferation of virulent bacteria in the lower airways. Alternatively, NTM bacteria may be easily suppressed early on in these patients, potentially reducing lung damage and maintaining a stable microbial environment. However, there are some limitations in our study that prevent us from reaching such a conclusion. Therefore, it is necessary to validate these findings in the future by incorporating a larger number of patients and employing more advanced sequencing techniques.

Interestingly, *Veillonella dispar*, *Fusobacterium periodonticum*, and *Pseudomonas aeruginosa* were previously mentioned in microbiome studies of diseases like lung cancer, chronic obstructive pulmonary disease, or sarcoidosis. Previous studies found that *Veillonella* in bronchoalveolar lavage fluid was associated with worsened non-small cell lung cancer or poor lung function of obstructive pulmonary disease in comparison to controls^19,20^. *Veillonella dispar* species are also observed in the oral cavity of smokers^21^. *Fusobacterium* was significantly more abundant in bronchoalveolar lavage samples from sarcoidosis patients than in those from healthy controls^22^, whereas *Fusobacterium* was reduced in nasopharyngeal swabs from COVID-19 patients^23^. *Pseudomonas aeruginosa* is linked to clinical deterioration in bronchiectasis patients, with dominance of *Pseudomonas* leading to greater disease severity, increased exacerbations, and higher mortality risk^24,25^. The BLESS study found that long-term erythromycin increased *Pseudomonas* proportions in sputum for patients without previous predominant *Pseudomonas* infection^13^. These phenomena indicate that certain microorganisms are involved in the development of various respiratory diseases. Based on our data, this topic also requires further clarification in NTM-PD with additional studies to explore the microbial environment that promotes NTM survival. Additionally, further investigations into the presence of bacterial antagonism or cooperation between NTM and other microorganisms during the treatment process of NTM-PD will be required.

In our study, we observed a decrease in the Lachnospiraceae family in the sputum of patients who used antibiotics. Lachnospiraceae is a bacterial family commonly found in the human gut microbiota, and it plays a crucial role in maintaining gut health and providing various benefits^26,27^. In recent studies using fecal microbiome analysis, Lachnospiraceae was reduced in patients with chronic obstructive pulmonary disease or lung cancer compared to normal subjects^28,29^. Considering the gut-lung axis effects and the potential human benefits of Lachnospiraceae, the observed decrease in Lachnospiraceae in our study may not be beneficial to NTM-PD patients, suggesting the need for antibiotics that cause less dysbiosis. However, drawing such a conclusion from our study alone is impossible, and we believe that further research in this field is necessary.

Our study had several limitations. Firstly, the number of patients included in this study was small, especially in the refractory group. Although sputum was collected from numerous patients prior to this study, the number of patients who consented to ongoing sputum collection was limited. Secondly, there are limitations in using 16S rRNA gene sequencing for the identification of mycobacteria. Previous studies have reported an inability to identify mycobacteria, including in positive culture samples for these organisms. Additionally, this method does not allow for the differentiation between *M. tuberculosis* and NTM, nor does it enable detailed species or subspecies identification of NTM. Moreover, there is a possibility of unidentified taxa when using 16S rRNA gene sequencing. Third, this study was conducted only on Asian patients treated at a single institution. Fourth, the majority of our study patients are female and had the radiological phenotype of nodular bronchiectatic form, which could have influenced the distribution of respiratory microbiomes. Lastly, because we did not use induced sputum, we cannot eliminate the influence of salivary contamination. However, we visually confirmed sufficient specimens during sputum collection and only used specimens where NTM was cultured from the samples collected for microbiome research.

Sputum microbiota diversity is reduced during treatment in NTM-PD patients. As treatment progresses, most taxa decreased in the culture conversion group, while certain taxa increased in the refractory group. These findings suggest a distinct microbial environment associated with refractory disease.

## Materials and methods

### Study participants

**Figure 4:**
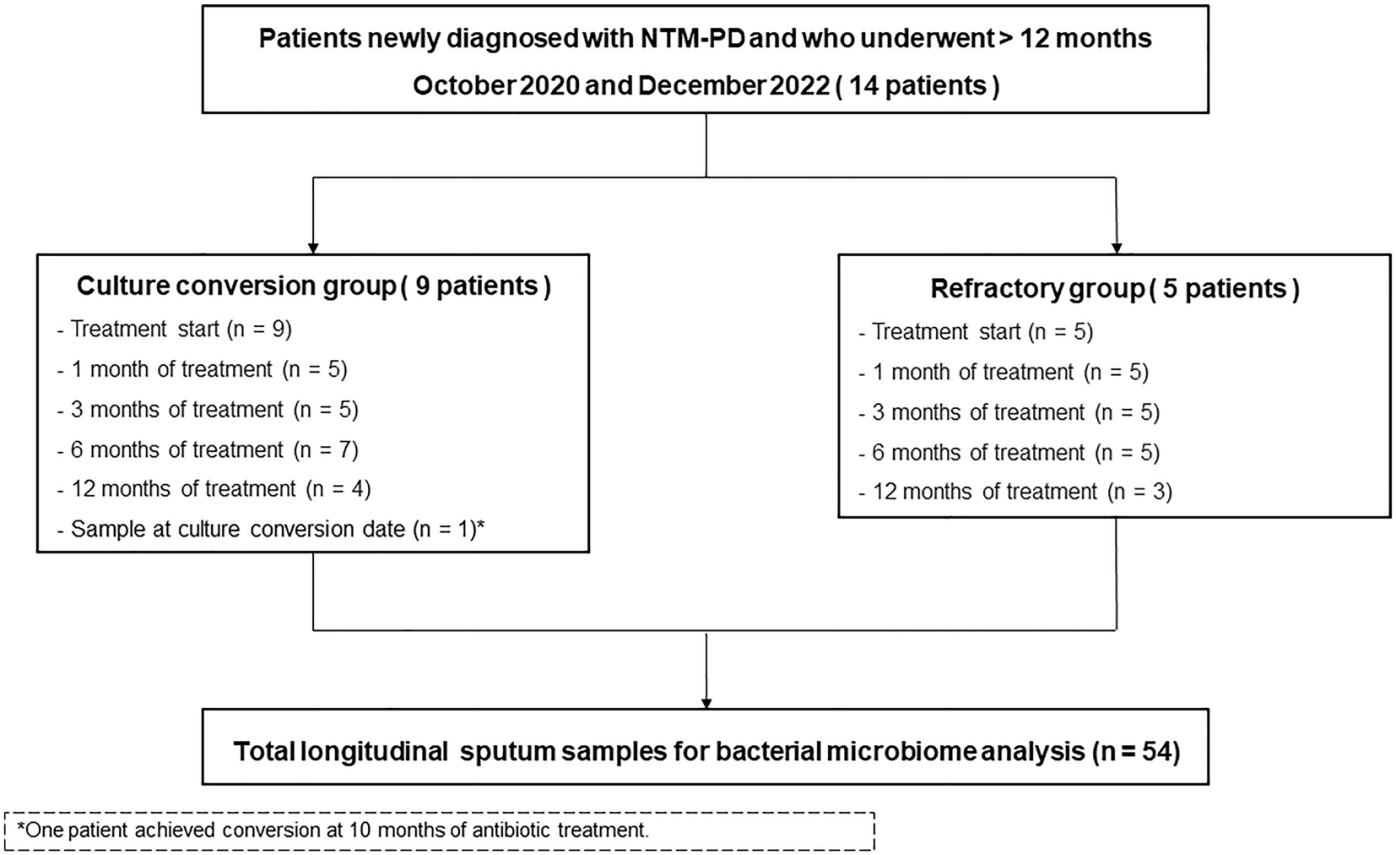
Flow chart of study groups.

From October 2020 to December 2022, among patients newly diagnosed with NTM-PD without a history of previous treatment, individuals who agreed to the collection of sputum samples and participation in the study were screened. All patients exhibited respiratory symptoms, radiological evidence of bronchiectasis or cavity lesions, and had positive NTM cultures from two separate sputum samples. All patients met the diagnostic criteria recommended in the clinical guidelines^4,7^. Among them, a total of 14 patients, whose sputum samples were collected during a 12-month course of antibiotic treatment, and whose response to NTM-PD treatment could be evaluated, were included in this study. Sputum samples were collected at specific time intervals: at the start of treatment and at 1 month, 3 months, 6 months, and 12 months after the initiation of antibiotics. Additionally, all patients were divided into two groups: the conversion group (n = 9), where negative culture conversion was achieved within 12 months of treatment, and the refractory group (n = 5), where persistent bacterial culture was observed despite antibiotic treatment. For subgroup analysis, sputum samples were also collected on the day of negative culture conversion in the conversion group; one patient achieved conversion on a different day from the regular sputum collection day (10-months of treatment). Consequently, a total of 54 sputum samples were collected from all 14 patients in the study, and the changes in the bacterial microbiome during treatment were analyzed (Fig. 4). Written informed consent was obtained from the study participants, and the Institutional Review Board of the Samsung Medical Center approved this study (IRB no. 2012-05-001). All methods were performed in accordance with the relevant guidelines and regulations.

### Sputum specimens

Approximately 3 ml of sputum was collected from the study patients. To minimize contamination, sputum was collected in the morning before eating any food, and for the collection, patients coughed deeply into a container that does not leak. The collected sputum was stored in a deep-freezer at − 80 °C, and microbiome analysis was performed within 3 days.

### DNA extraction from sputum and MiSeq sequencing

DNA extraction from sputum was performed using MP Biomedicals FastDNA® Spin Kit for soil (MPbio, Santa Ana, CA, USA) according to the manufacturer's protocol. Then, the concentration and quality of the extracted DNA were measured using the Epoch™ Spectrometer (BioTek, Winooski, VT, USA). DNA concentration was measured with the following guidelines: DNA concentration > 15 ng/µl, volume > 20 µl, A260/A280 ratio: 1.8–2.0. The library product was measured using the Bioanalyzer 2100, with these guidelines: Library product concentration > 12 ng/µl, volume > 20 µl, product size: 400–600 bp. Library product band size was confirmed via agarose gel electrophoresis. In order to analyze the bacterial microbiome in sputum, the V3-V4 region of the 16S rRNA gene in bacteria was amplified^18^.

Based on the MiSeq system protocol for preparing a 16S metagenomics sequencing library, a second PCR (index PCR) was performed to attach an index sequence and an Illumina sequencing adapter to the PCR product using the Nextera XT DNA Library Preparation Kit (Illumina, San Diego, CA, USA). The second PCR-completed product was purified using the QIAquick PCR purification kit (Qiagen, Valencia, CA, USA) and quantified using the Quanti-iT PicoGreen dsDNA Assay Kit (Invitrogen, Waltham, MA, USA). The quality of the final library product was measured using the Bioanalyzer 2100 (Agilent, Palo Alto, CA, USA). Samples that did not pass quality control during the DNA extraction and library production steps were excluded from the experiment. The library products that passed quality control were sequenced at CJ Bioscience, Inc. (Seoul, Korea) according to the manufacturer's instructions using the MiSeq Reagent Kit v2 (500-cycles) based on the Illumina MiSeq sequencing platform (Illumina, San Diego, CA, USA).

### Sequence analysis

Microbiome profiling was performed using the 16S-based Microbial Taxonomic Profiling (MTP) platform of the EzBioCloud application, which uses the 16S database version PKSSU.4.0^30^. All raw sequencing data were re-analyzed into individual MTPs using the EzBioCloud pipeline. MTP sets were constructed by grouping these individual MTPs, and comparative analysis between MTP sets was performed after normalization of gene copy numbers. The 16S gene copy number of bacteria varies by up to 15 times depending on the species. Sequencing reads obtained from 16S rRNA sequence amplification products are classified into species for which the 16S rRNA sequence is known, and the number of reads is used to calculate relative species abundance. The EzBioCloud application we used provides 16S gene copy number information for over 3200 species and normalizes the 16S gene copy number for each analyzed bacterial species based on this information.

The relative abundance of sequences was compared between MTP sets using the Wilcoxon rank-sum test. Alpha-diversity was analyzed using the species richness (ACE, Chao1, Jackknife, and number of OTU), while beta-diversity was evaluated using generalized UniFrac distances and visualized by principal component analysis. Permutational multivariate analysis of variance was used to analyze statistical differences in beta diversity. To identify differentially distributed taxa between MTP sets, LDA was performed to evaluate different groups based on the LEfSe. LEfSe is a method that uses LDA to assess the effect size of discriminating characteristics between groups. LDA transforms data to maximize group average differences. It quantifies effect size with the LDA score, combining group mean difference and trait variance. Higher LDA scores indicate greater importance in group distinction. We computed LDA scores for each group in LEfSe analysis. Statistical significance was set at *p* < 0.05. Hotelling’s t-test was used to compare bacterial profiles among categories.

## Ethical approval

Data were obtained from an ongoing prospective observational cohort (ClinicalTrials.gov identifier: NCT00970801), and the Institutional Review Board of the Samsung Medical Center approved this study (IRB no. 2012–05–001). We obtained the written consent from the participants.

### Supplementary Information

Supplementary Information.

## Data Availability

Raw sequencing data have been deposited in the Sequence Read Archive website (Reference number PRJNA1005347).
