# Supplementary material for: Changes in sputum microbiota during treatment for nontuberculous mycobacterial pulmonary disease
Source: Sci Rep. 2023 Nov 13;13:19764. doi: 10.1038/s41598-023-47230-5 (PMC10643529; doi:10.1038/s41598-023-47230-5)

**[Online supplementary data]**

**Changes in Sputum Microbiota during Treatment for Nontuberculous Mycobacterial Pulmonary Disease**

Bo-Guen Kim^1¶^, Jin Young Yu^2¶^, Su-Young Kim^3^, Dae Hun Kim^3*^, Byung Woo Jhun^3*^

^1^Division of Pulmonary Medicine and Allergy, Department of Internal Medicine, Hanyang University College of Medicine, Seoul, Korea

^2^Department of Medicine, Samsung Medical Center, Sungkyunkwan University School of Medicine, Seoul, South Korea

^3^Division of Pulmonary and Critical Care Medicine, Department of Medicine, Samsung Medical Center, Sungkyunkwan University School of Medicine, Seoul, South Korea

**Correspondence to**: Byung Woo Jhun, Division of Pulmonary and Critical Care Medicine, Department of Medicine, Samsung Medical Center, Sungkyunkwan University School of Medicine, 81 Irwon-ro, Gangnam-gu, Seoul, 06351, South of Korea.

E-mail: [byungwoo.jhun@gmail.com](mailto:byungwoo.jhun@gmail.com)

^¶^These authors (Bo-Guen Kim and Jin Young Yu) contributed equally to this work

^*^Co-senior authors

**Table S1**. Sputum collection dates during antibiotic therapy in study patients

| **Patients** | **Etiology** | **Sputum collection dates** | | | | | |
| --- | --- | --- | --- | --- | --- | --- | --- |
|  |  | **Antibiotics start** | **1 mo.** | **3 mo.** | **6 mo.** | **12 mo.** | **Culture**  **conversion date^¶^** |
| Refractory 1 | *M. intracellulare* | **√** | **√** | **√** | **√** | **√** | - |
| Refractory 2 | *M. intracellulare/*  *M. abscessus* | **√** | **√** | **√** | **√** | **√** | - |
| Refractory 3 | *M. intracellulare* | **√** | **√** | **√** | **√** | - | - |
| Refractory 4 | *M. massiliense* | **√** | **√** | **√** | **√** | **√** | - |
| Refractory 5 | *M. massiliense* | **√** | **√** | **√** | **√** | - | - |
| Conversion 6 | *M. intracellulare* | **√** | **√** | **√** | **√** | - | **√**  (1 mo. after therapy) |
| Conversion 7 | *M. avium* | **√** | - | **√** | **√** | **√** | **√**  (3 mo. after therapy) |
| Conversion 8 | *M. avium* | **√** | **√** | - | **√** | **√** | **√**  (12 mo. after therapy) |
| Conversion 9 | *M. avium/*  *M. abscessus* | **√** | - | - | - | - | **√**  (10 mo. after therapy) |
| Conversion 10 | *M. intracellulare* | **√** | - | - | **√** | - | **√**  (6 mo. after therapy) |
| Conversion 11 | *M. avium/*  *M. intracellulare* | **√** | **√** | - | - | - | **√**  (1 mo. after therapy) |
| Conversion 12 | *M. intracellulare* | **√** | - | **√** | **√** | **√** | **√**  (3 mo. after therapy) |
| Conversion 13 | *M. intracellulare* | **√** | **√** | **√** | **√** | **√** | **√**  (1 mo. after therapy) |
| Conversion 14 | *M. intracellulare* | **√** | **√** | **√** | **√** | - | **√**  (1 mo. after therapy) |

**^¶^**Sputum collected on the date of culture conversion. The gray shaded blocks indicate when antibiotic therapy was maintained.

**Table S2.** Details of antibiotics used in study patients

| **Antibiotics** | **Total (n = 14)** | **Conversion (n = 9)** | **Refractory (n = 5)** |
| --- | --- | --- | --- |
| Macrolide | 14 (100) | 9 (100) | 5 (100) |
| Duration, months | 12.7 (11.4 – 14.1) | 12.4 (10.0 – 13.8) | 13.8 (11.8 – 26.0) |
| Ethambutol | 11 (79) | 9 (100) | 2 (40) |
| Duration, months | 12.0 (9.0 – 13.2) | 12.0 (9.5 – 13.1) | 9.0, 23.1 |
| Rifampicin | 10 (71) | 8 (89) | 2 (40) |
| Duration, months | 12.0 (7.6 – 13.8) | 12.0 (8.4 –13.5) | 0.9, 13.8 |
| Fluoroquinolone | 3 (21) | 1 (11) | 2 (40) |
| Duration, months | 12.6 (0.9 – 13.8) | 12.6 | 0.9, 13.8 |
| Intravenous Amikacin | 3 (21) | - | 3 (60) |
| Duration, months | 5.1 (4.2 – 11.1) | - | 5.1 (4.2 – 11.1) |
| Inhaled amikacin | 3 (21) | - | 3 (60) |
| Duration, months | 7.8 (6.5 – 11.2) | - | 7.8 (6.5 – 11.2) |
| Imipenem | 3 (21) | - | 3 (60) |
| Duration, months | 1.8 (0.8 – 4.2) | - | 7.8 (6.5 – 11.2) |
| Tigecycline | 1 (7) | - | 1 (20) |
| Duration, months | 1.8 | - | 1.8 |
| Clofazimine | 3 (21) | - | 3 (60) |
| Duration, months | 3.3 (1.1 – 24.9) | - | 3.3 (1.1 – 24.9) |

Data are presented as number (%) or median (interquartile range).

**Figure S1.** Relative proportions of bacterial taxa during the course of antibiotic treatment in the all patients (phylum level).


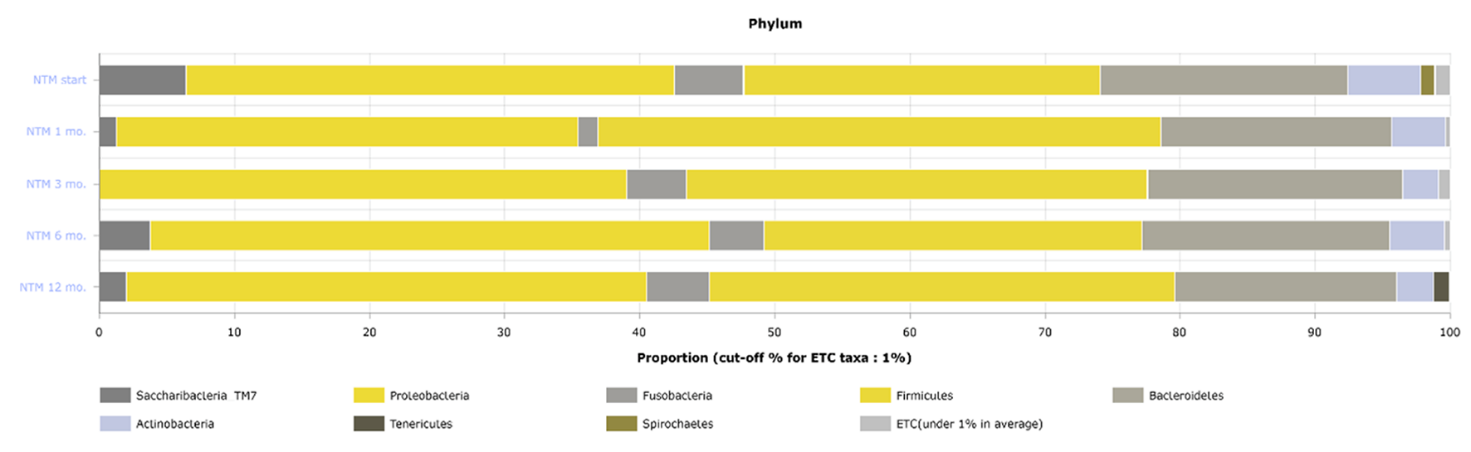


A) *Saccharibacteria* (NTM start *vs.* NTM 1 mo. p = 0.015; NTM start *vs.* NTM 3 mo. p = 0.002; Wilcoxon rank-sum test).


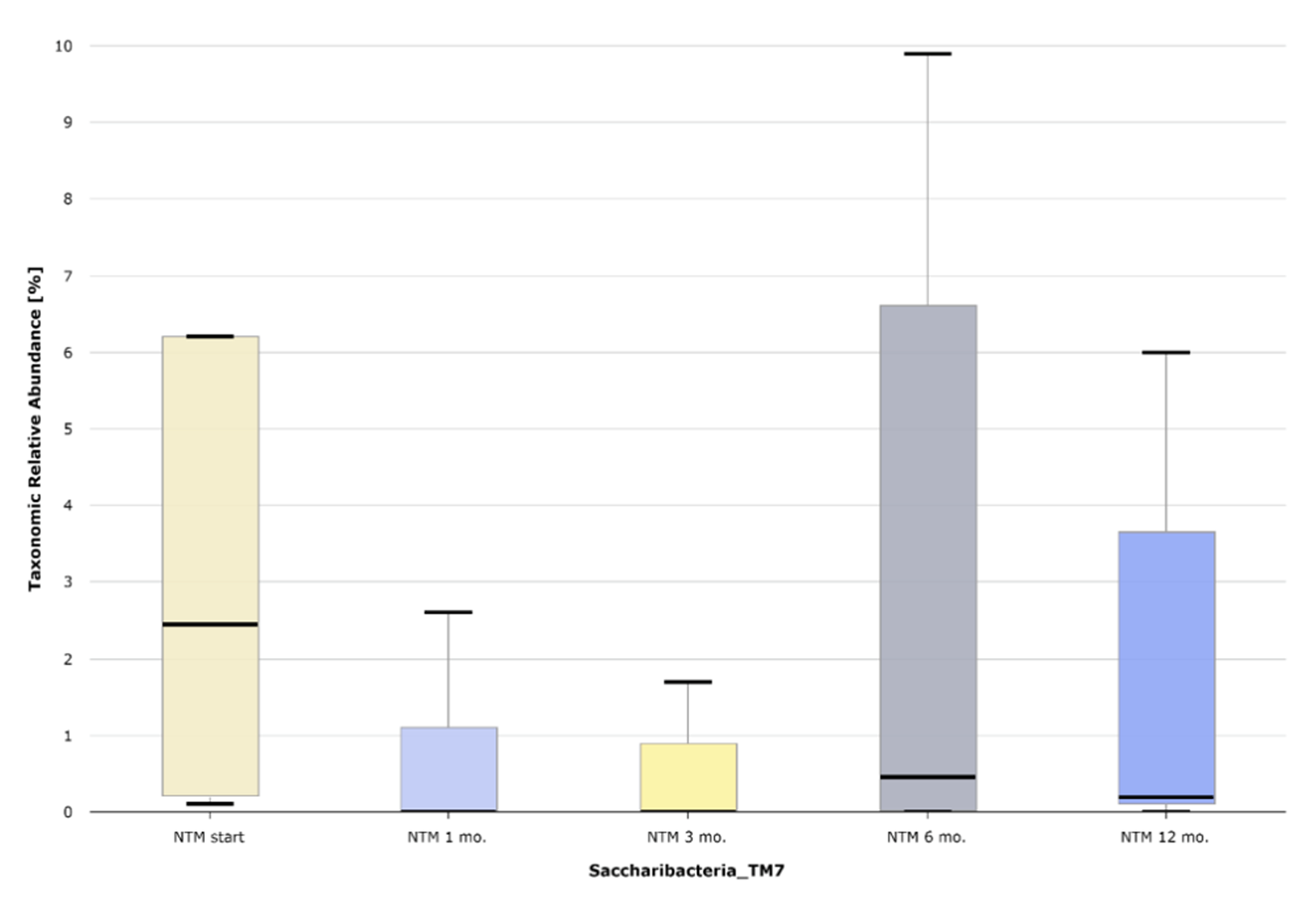


B) *Fusobacteria* (NTM start *vs.* NTM 1 mo. p = 0.008; NTM 1 mo. *vs.* NTM 12 mo. p = 0.025; Wilcoxon rank-sum test)


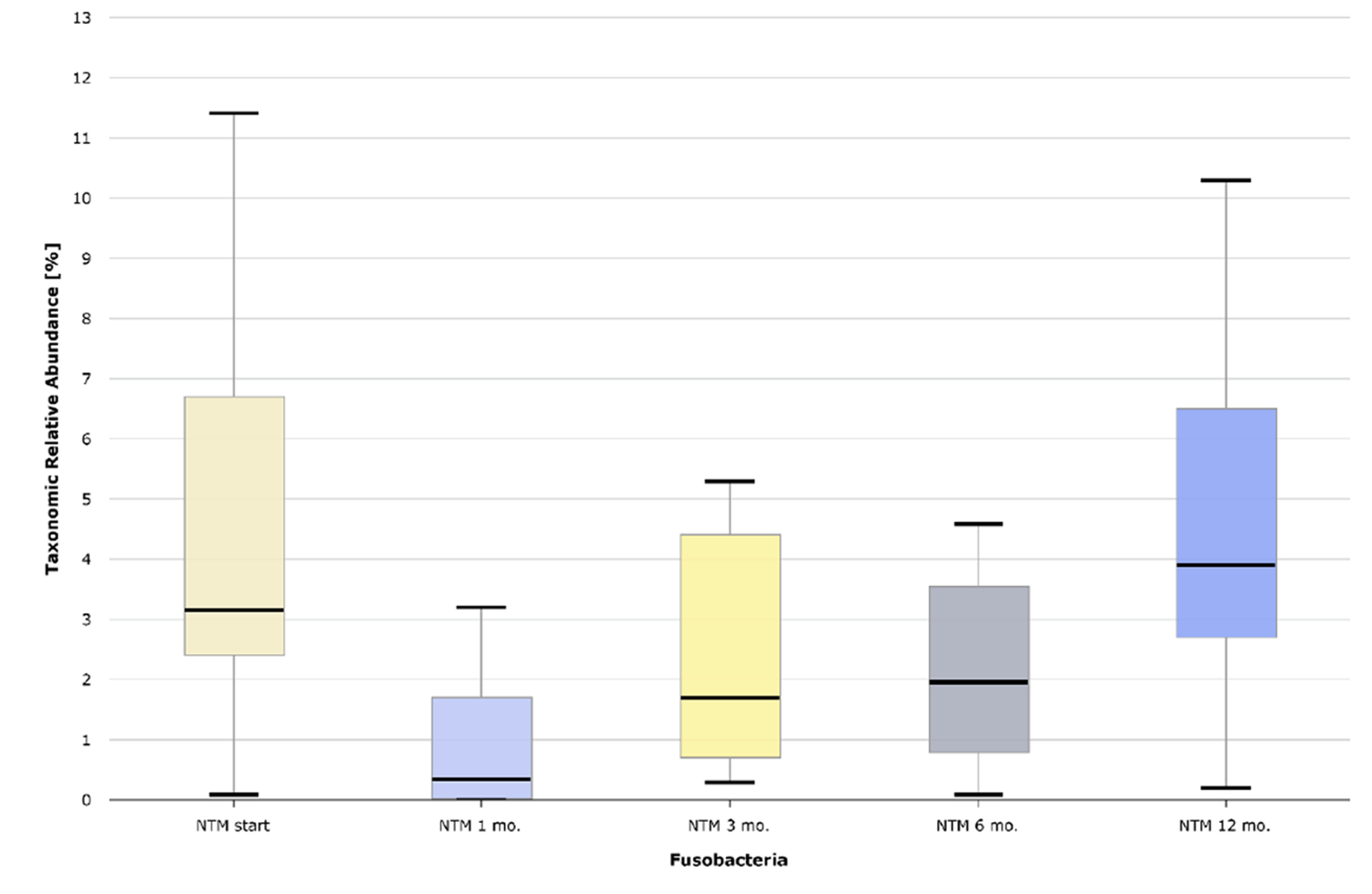


C) *Actinobacteria* (NTM start *vs.* NTM 3 mo. p = 0.022; NTM 1 mo. *vs.* NTM 6 mo. p = 0.040; Wilcoxon rank-sum test)


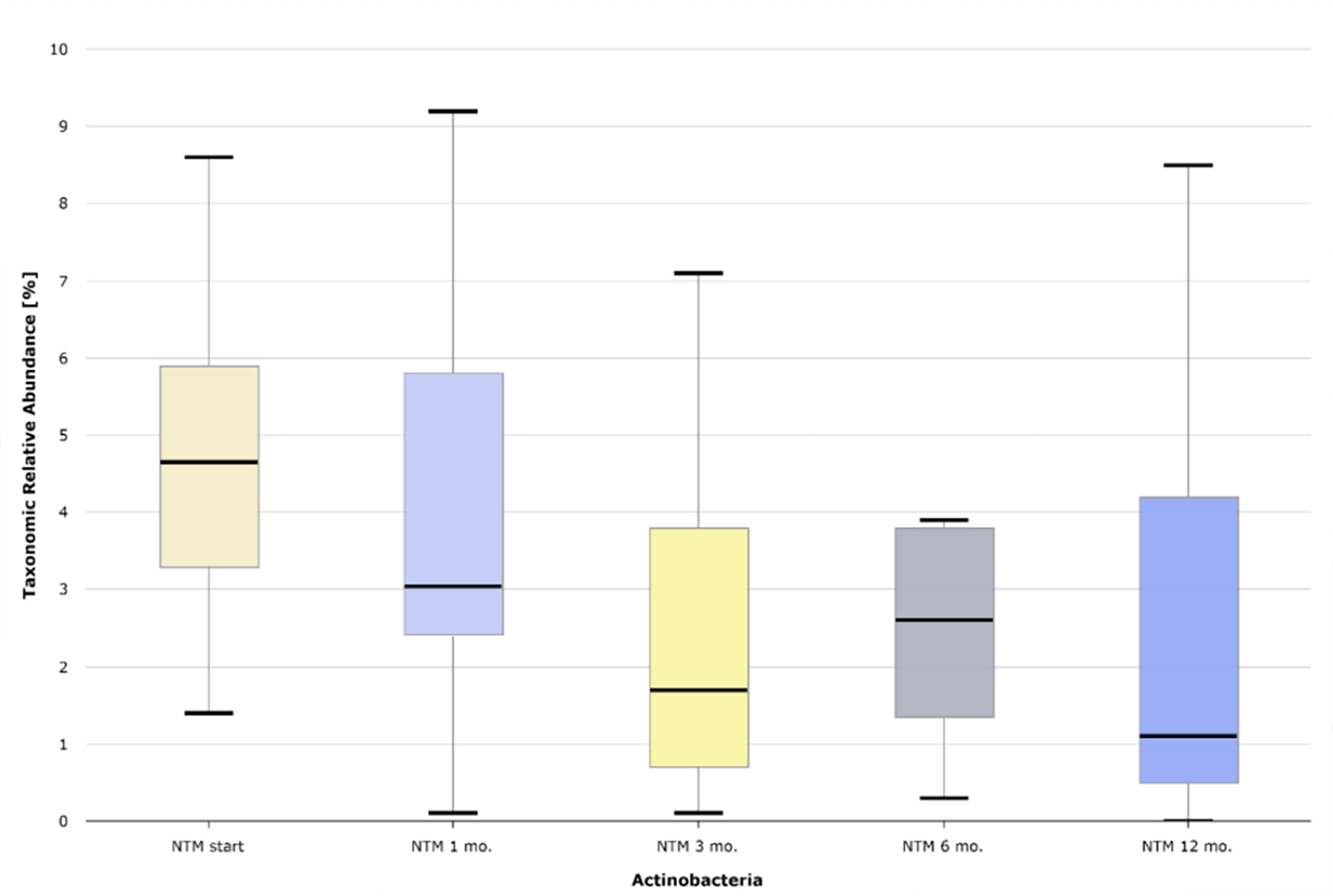


D) *Spirochaetes* (NTM start *vs.* NTM 1 mo. p <0.001; NTM start *vs.* NTM 3 mo. p = 0.001; NTM start *vs.* NTM 6 mo. p = 0.001; NTM start *vs.* NTM 12 mo. p = 0.003; Wilcoxon rank-sum test)
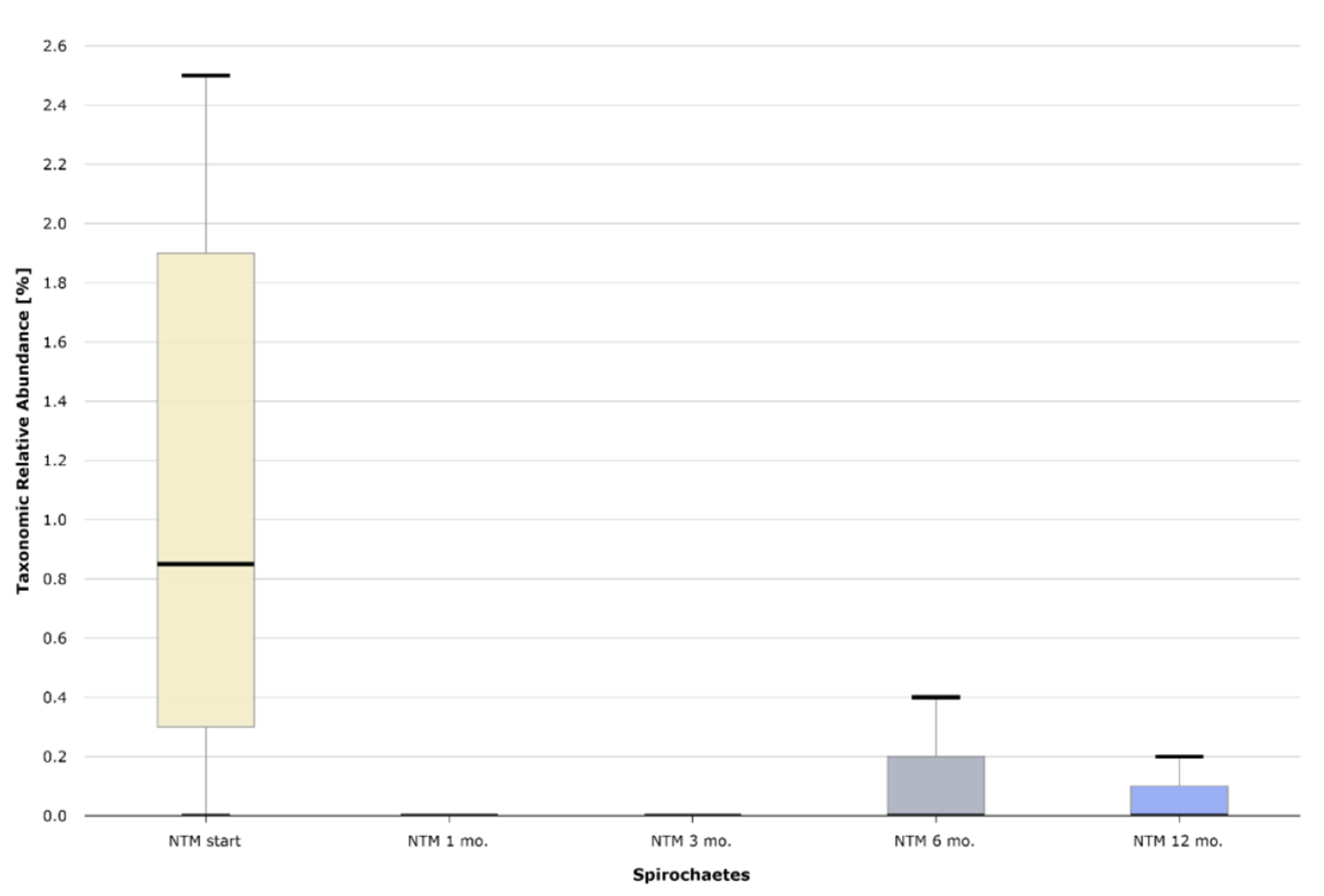


**Figure S2.** Relative proportions of bacterial taxa during the course of antibiotic treatment in the all patients (genus or family level).


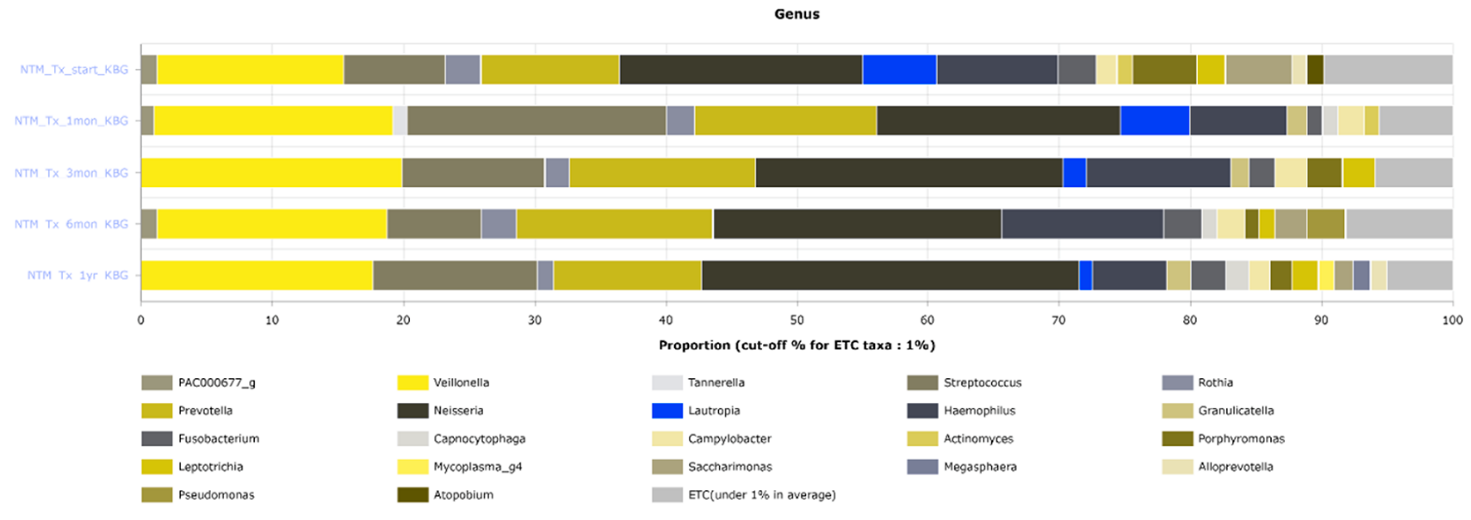


A) *Tannerella* (NTM start *vs.* NTM 3 mo. p = 0.012; Wilcoxon rank-sum test)


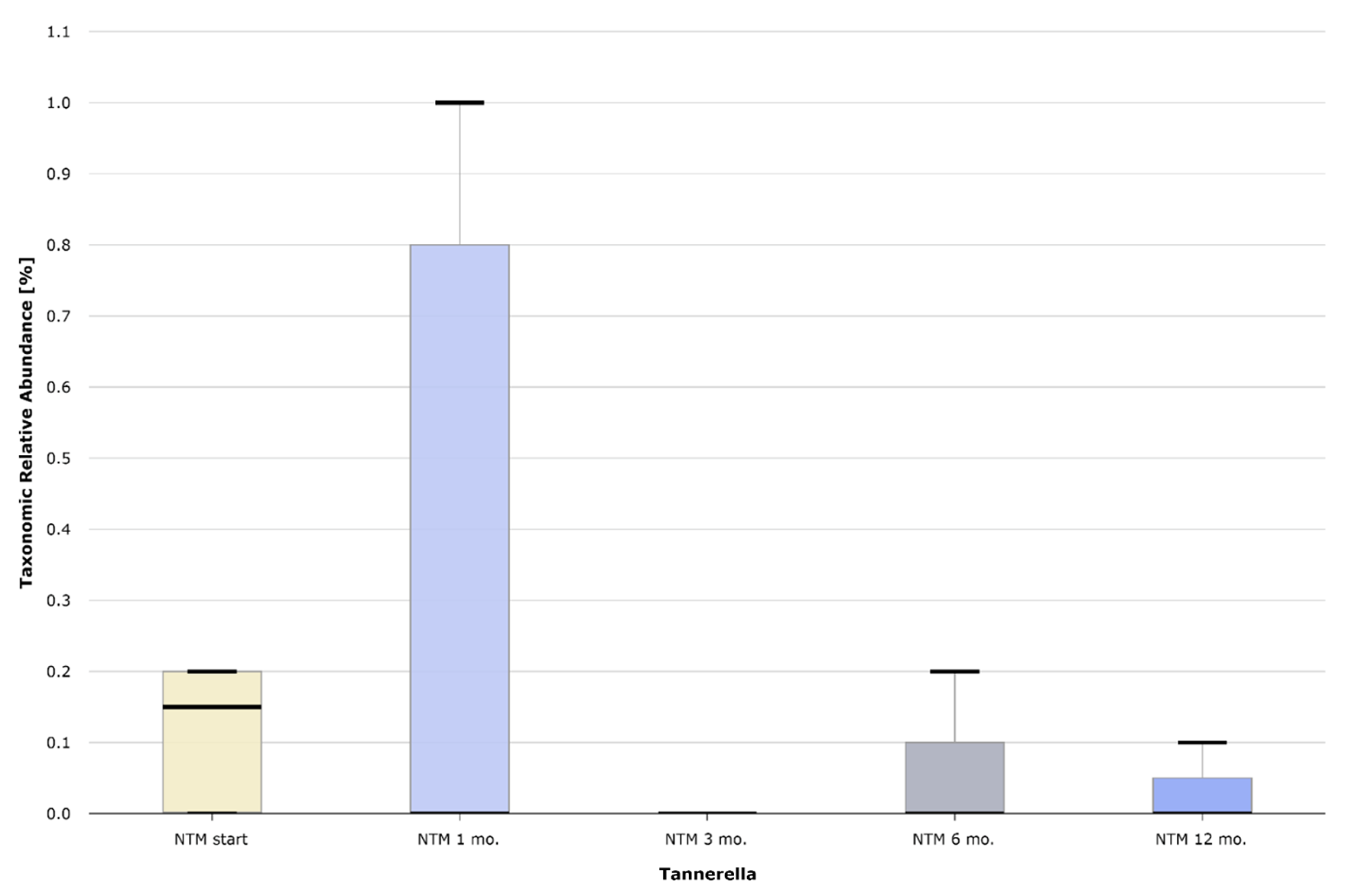


B) *Fusobacterium* (NTM start *vs.* NTM 1 mo. p = 0.006; NTM 1mo. *vs.* NTM 6 mo. p = 0.038; Wilcoxon rank-sum test)


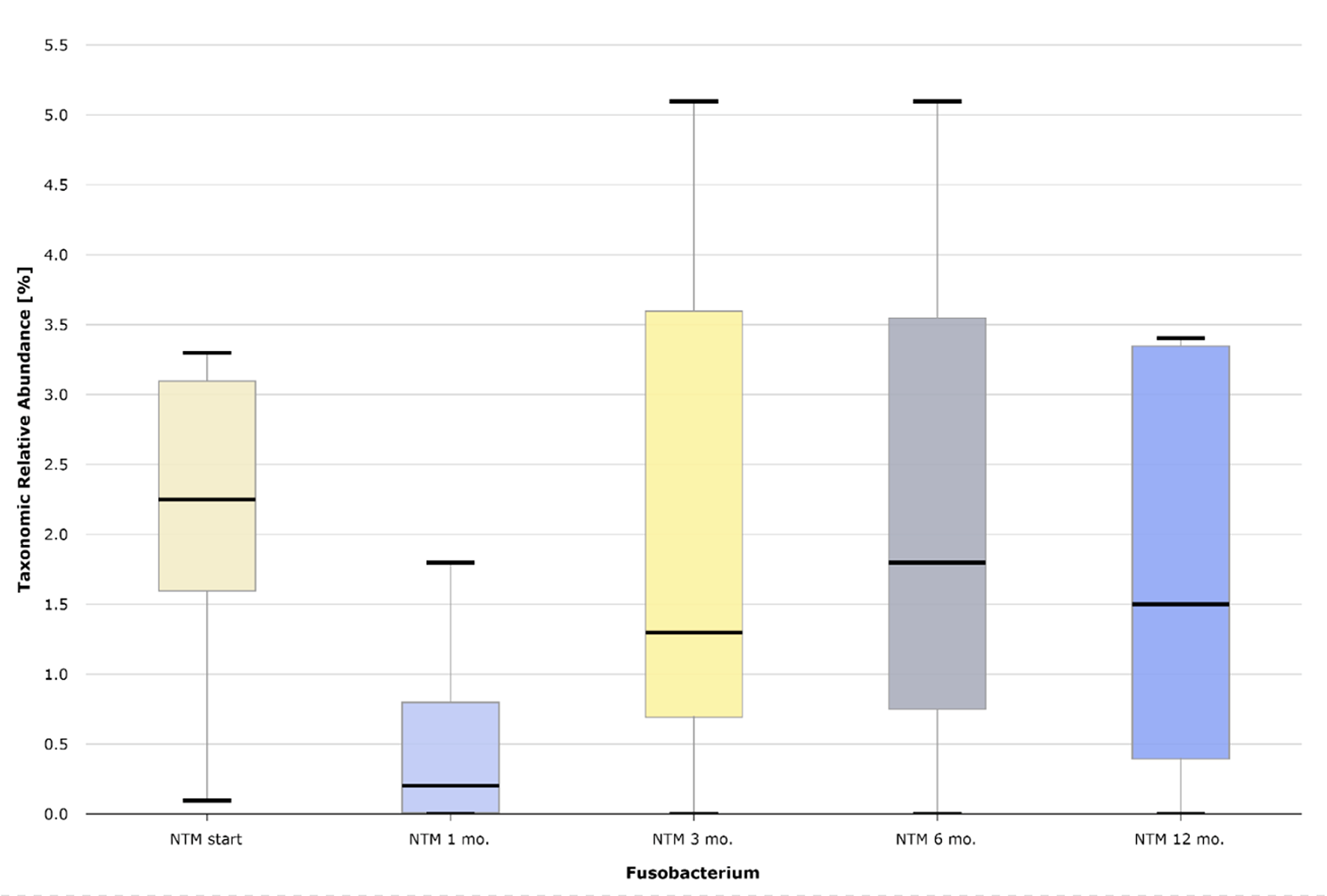


C) *Actinomyces* (NTM start *vs.* NTM 3 mo. p = 0.008; Wilcoxon rank-sum test)


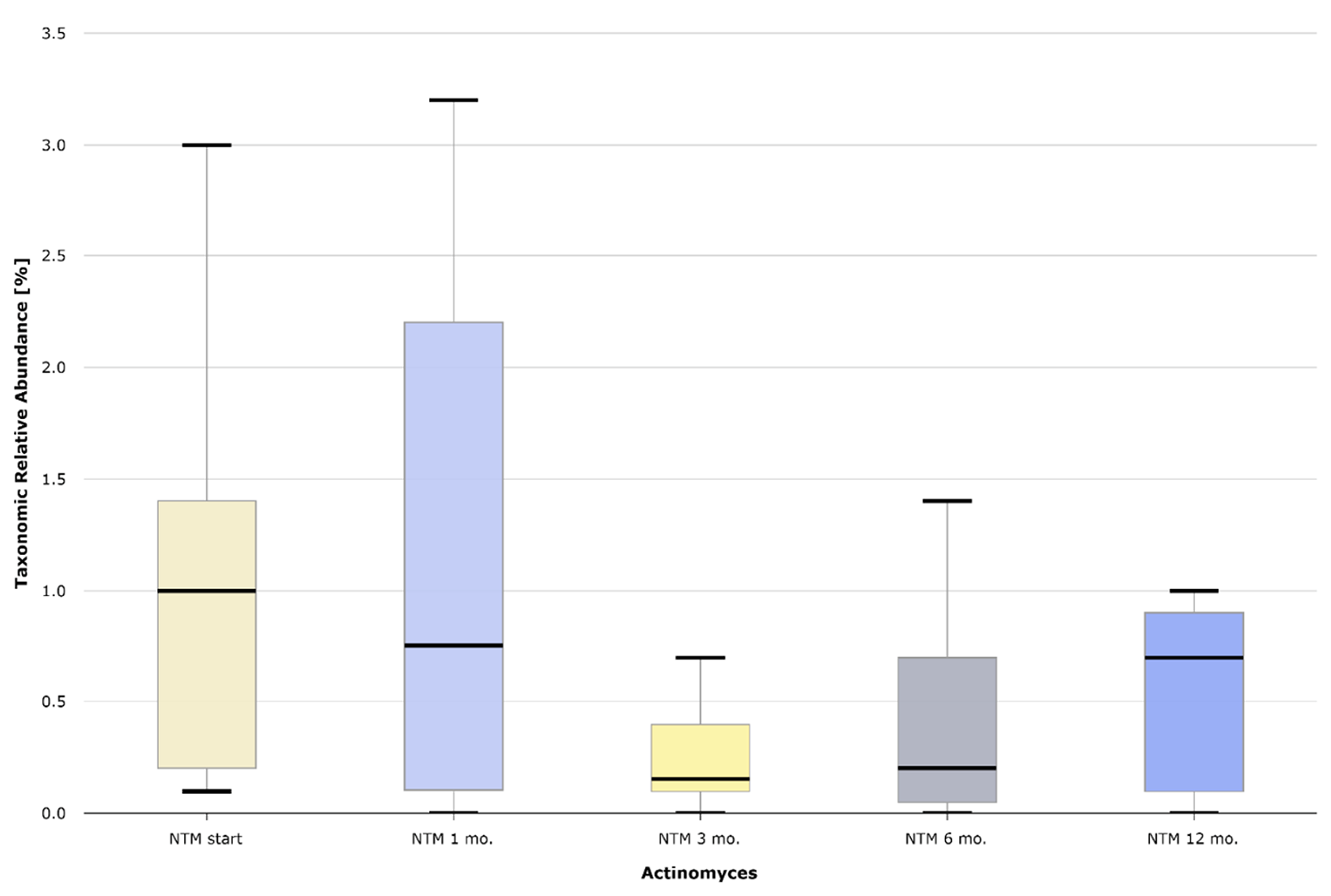


D) *Porphyromonas* (NTM start *vs.* NTM 1 mo. p = 0.004; NTM start *vs.* NTM 6 mo. p = 0.011; Wilcoxon rank-sum test)


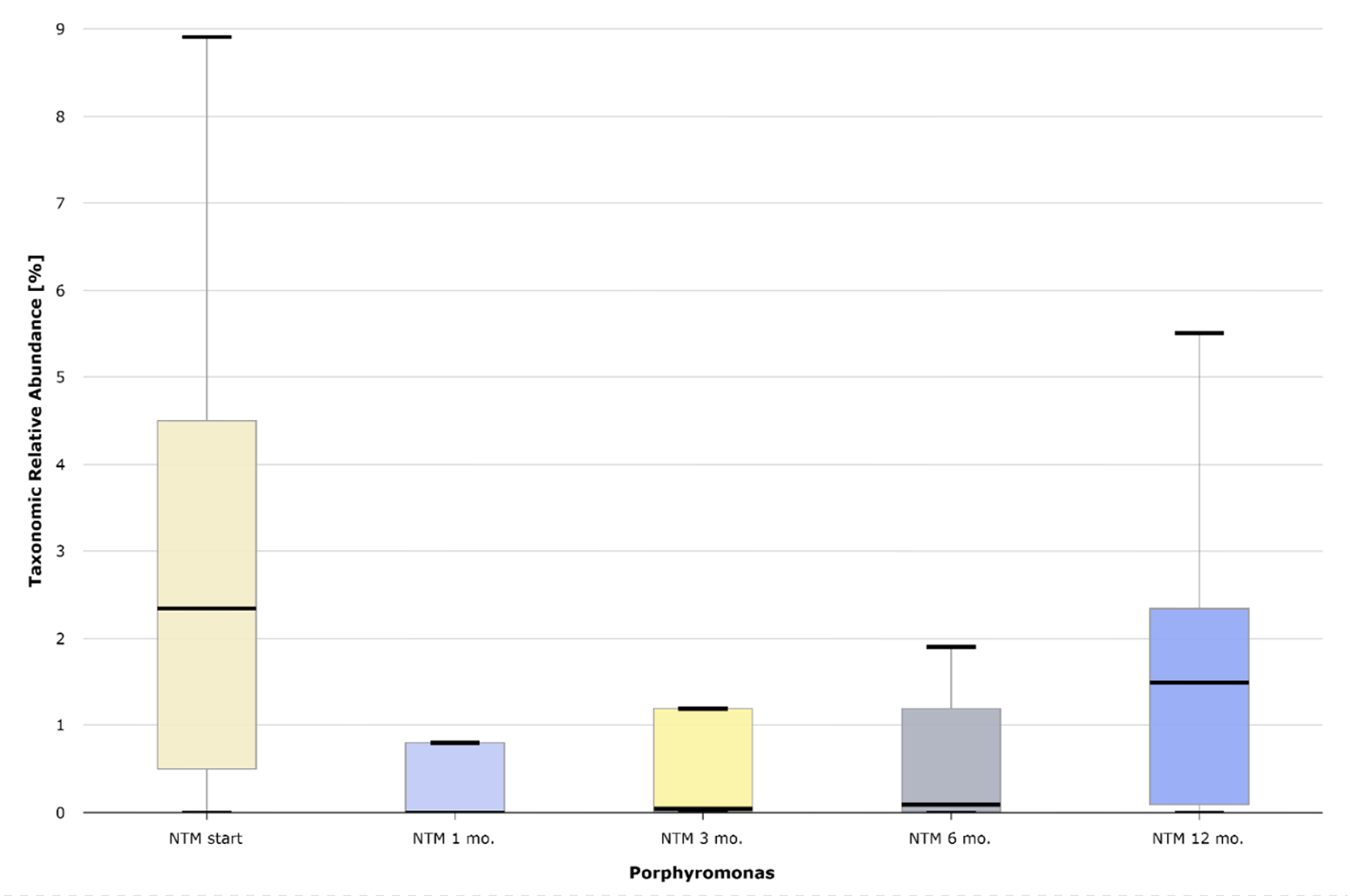


E) *Saccharimonas* (NTM start *vs.* NTM 1 mo. p = 0.002; NTM start *vs.* NTM 3 mo. p = 0.001; NTM start *vs.* NTM 6 mo. p = 0.031; NTM 3 mo. *vs.* NTM 12 mo. p = 0.0.32; Wilcoxon rank-sum test)


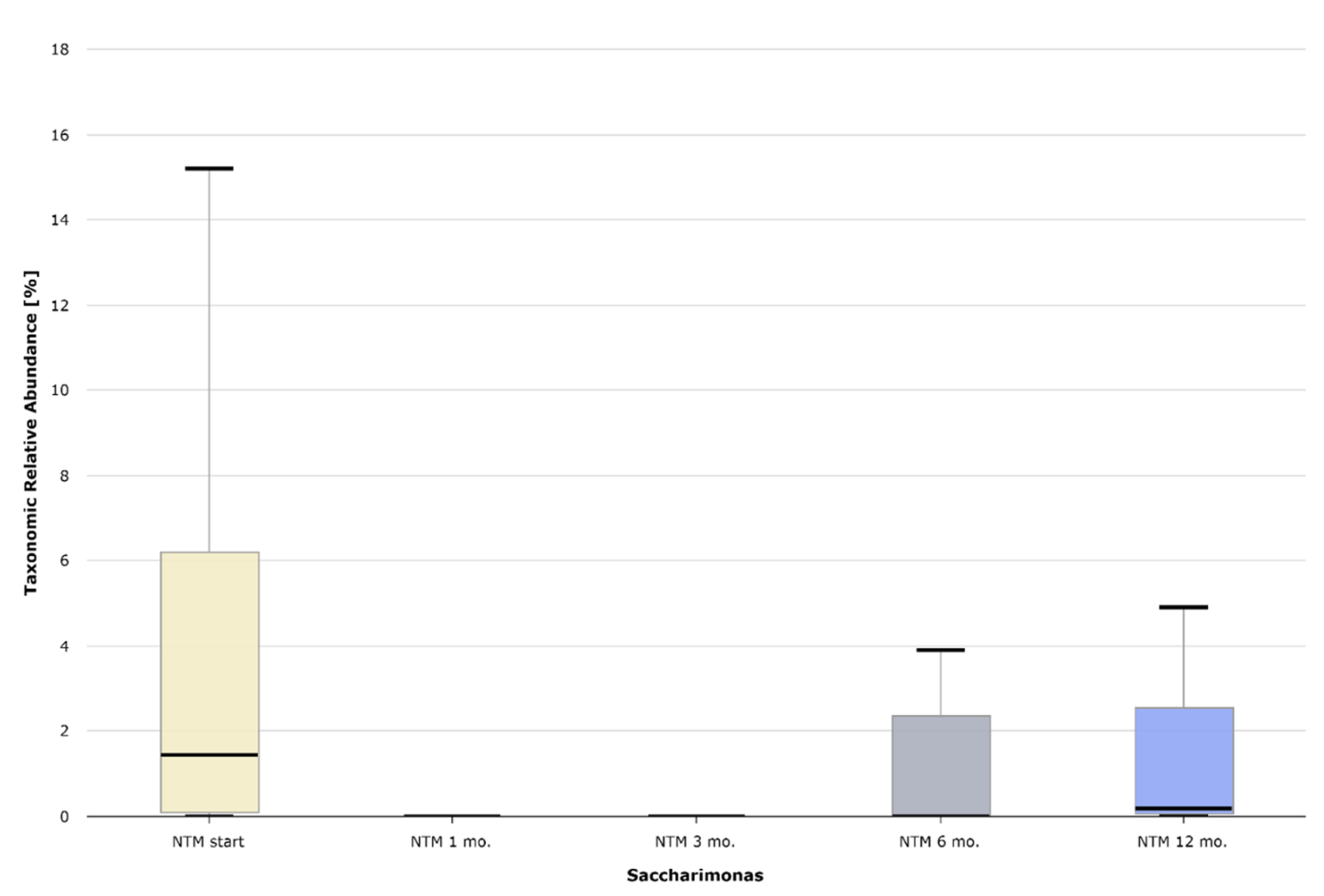


F) *Alloprevotella* (NTM start *vs.* NTM 1 mo. p = 0.007; Wilcoxon rank-sum test)


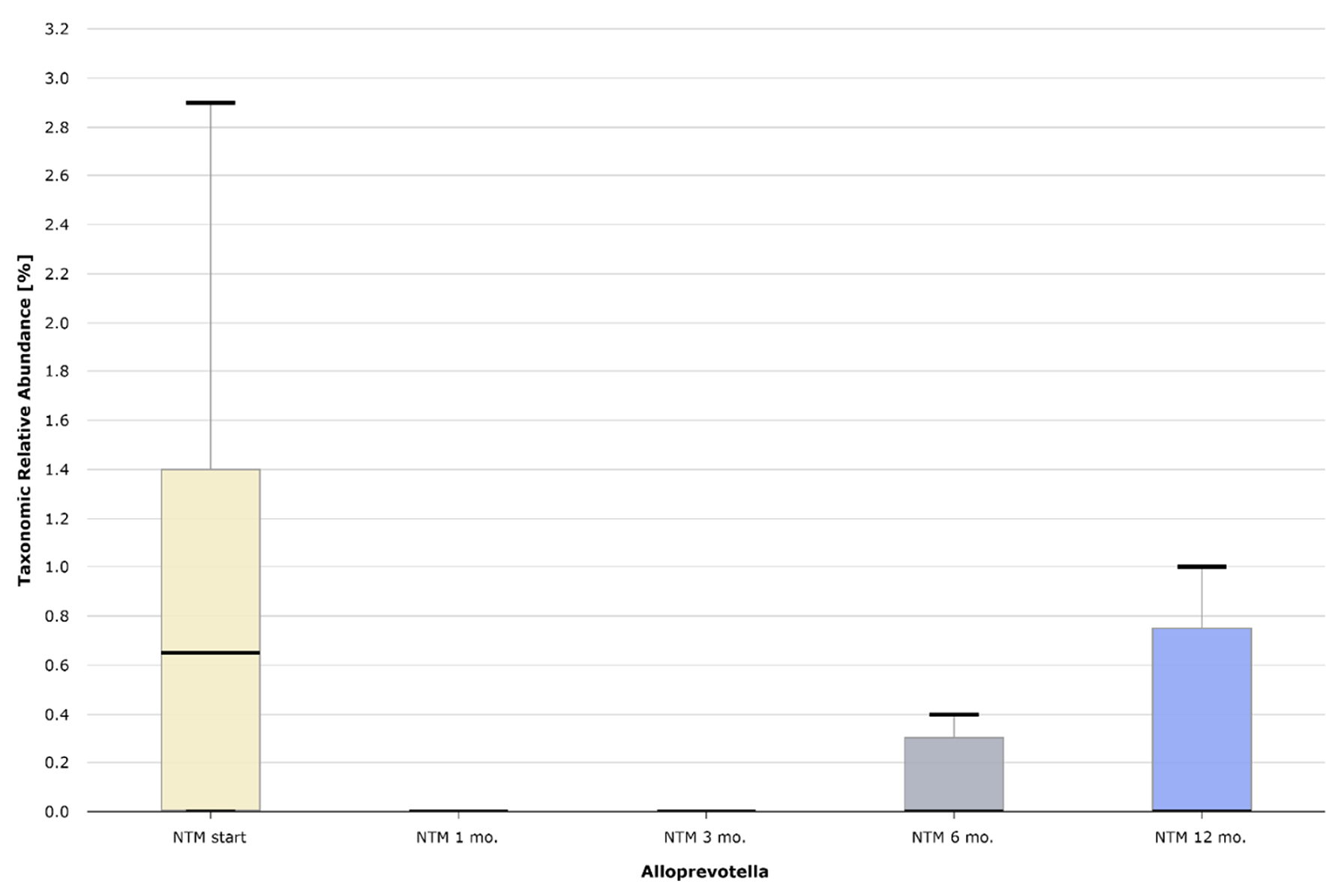


G) *Pseudomonas*


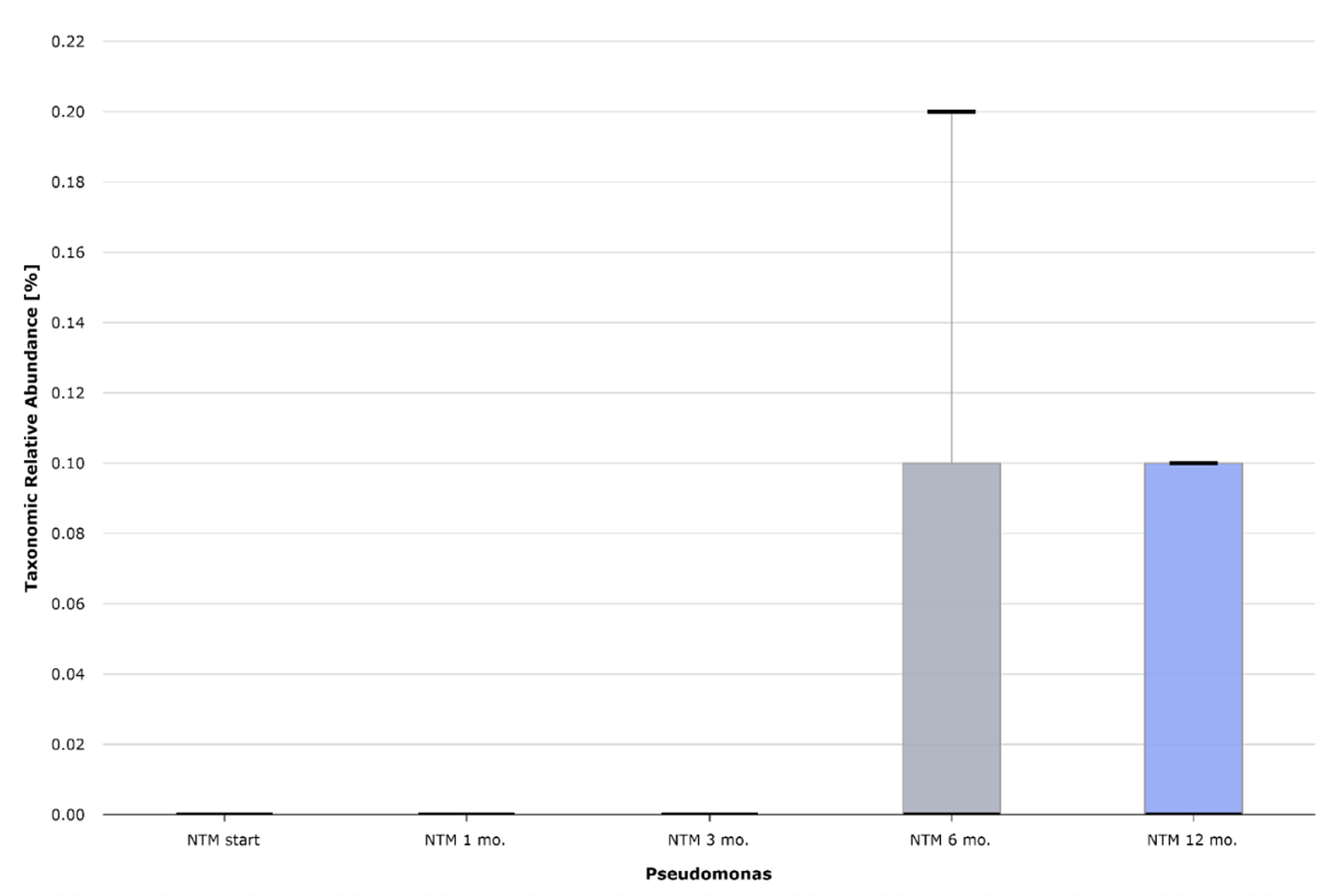


F) *Lachnospiraceae*, family (NTM start *vs.* NTM 3 mo. p = 0.038; Wilcoxon rank-sum test)


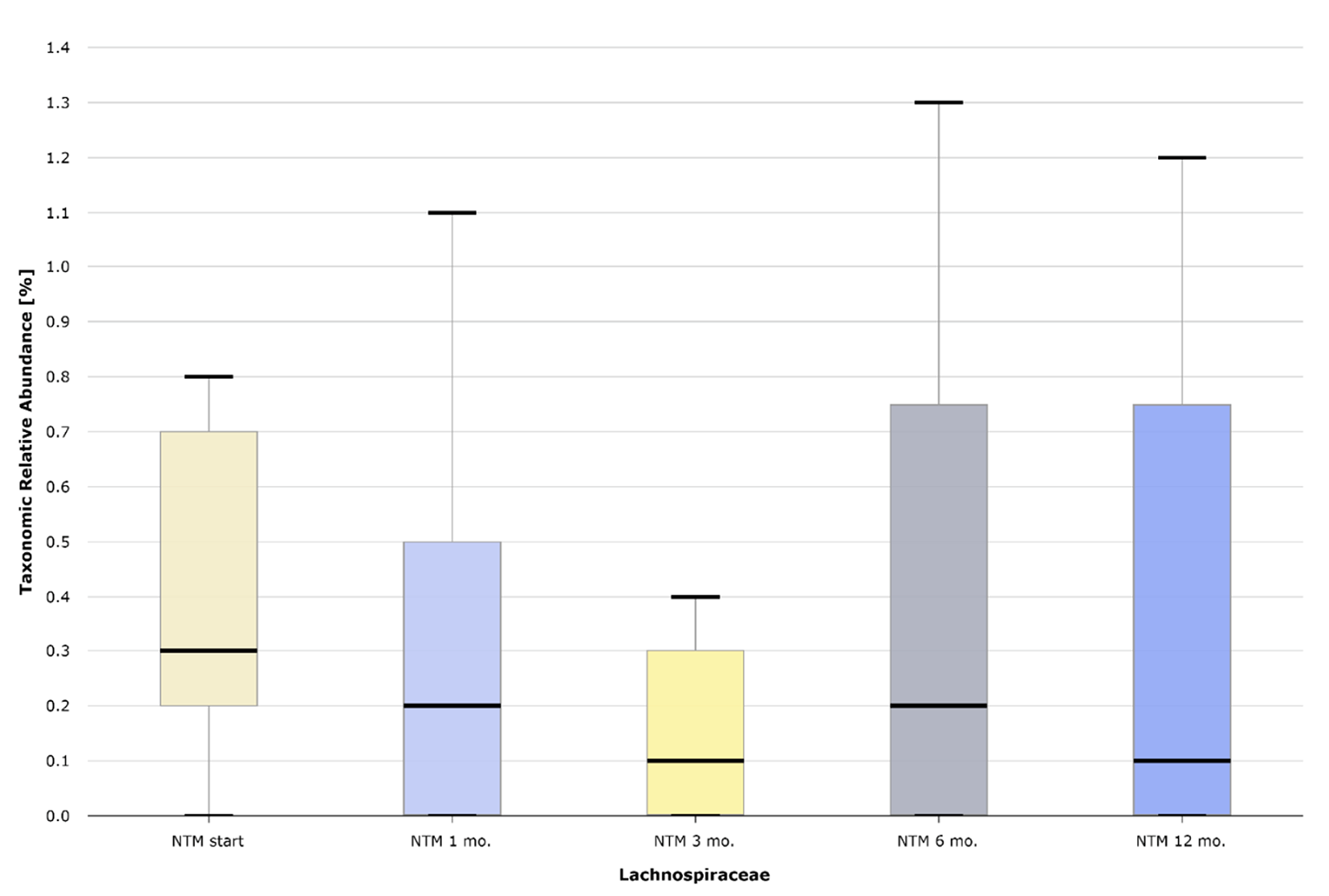


**Figure S3**. Relative proportions of bacterial taxa during the course of antibiotic treatment in the treatment refractory group (genus level).

(A) *Veillonella dispar* (start *vs.* 1 mo. p = 0.009; start *vs.* 3 mo. p = 0.009; start *vs.* 6 mo. p = 0.025; Wilcoxon rank-sum test)


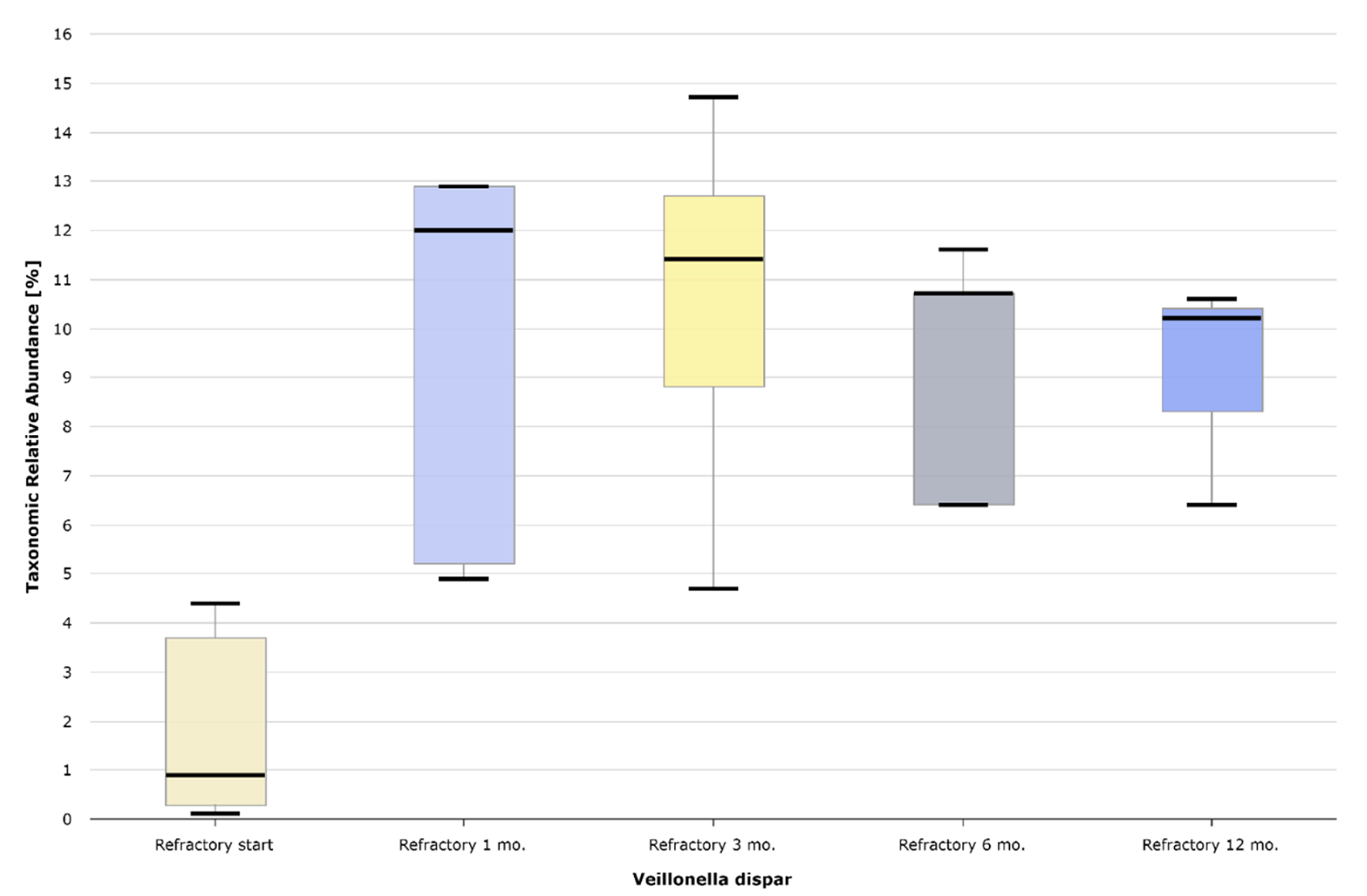


(B) *Fusobacterium periodonticum* (1 mo. *vs.* 3 mo. p = 0.047; 1 mo. *vs.* 6 mo. p = 0.047; 1 mo. *vs.* 12 mo. p = 0.025; Wilcoxon rank-sum test)


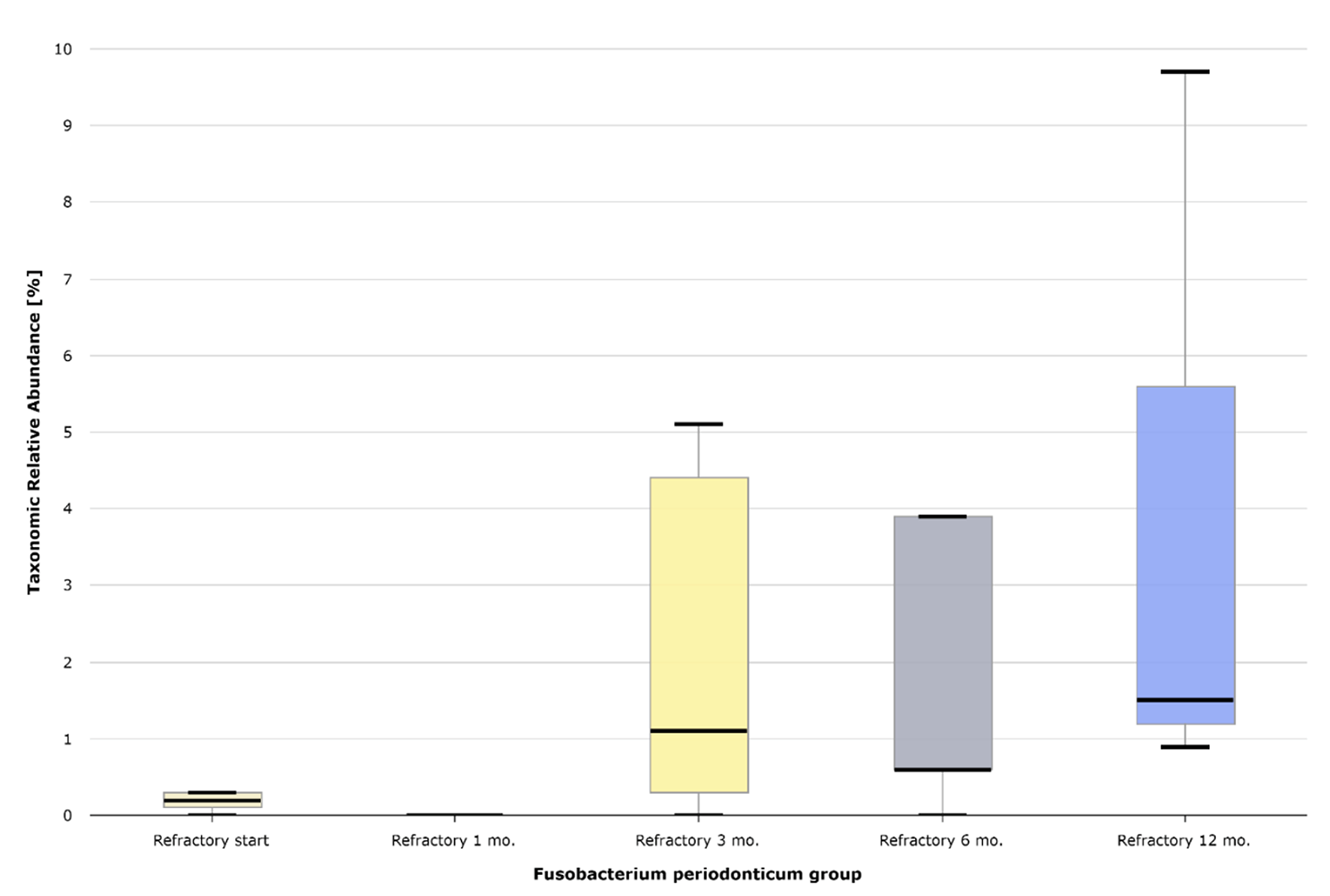


(C) *Pseudomonas aeruginosa*


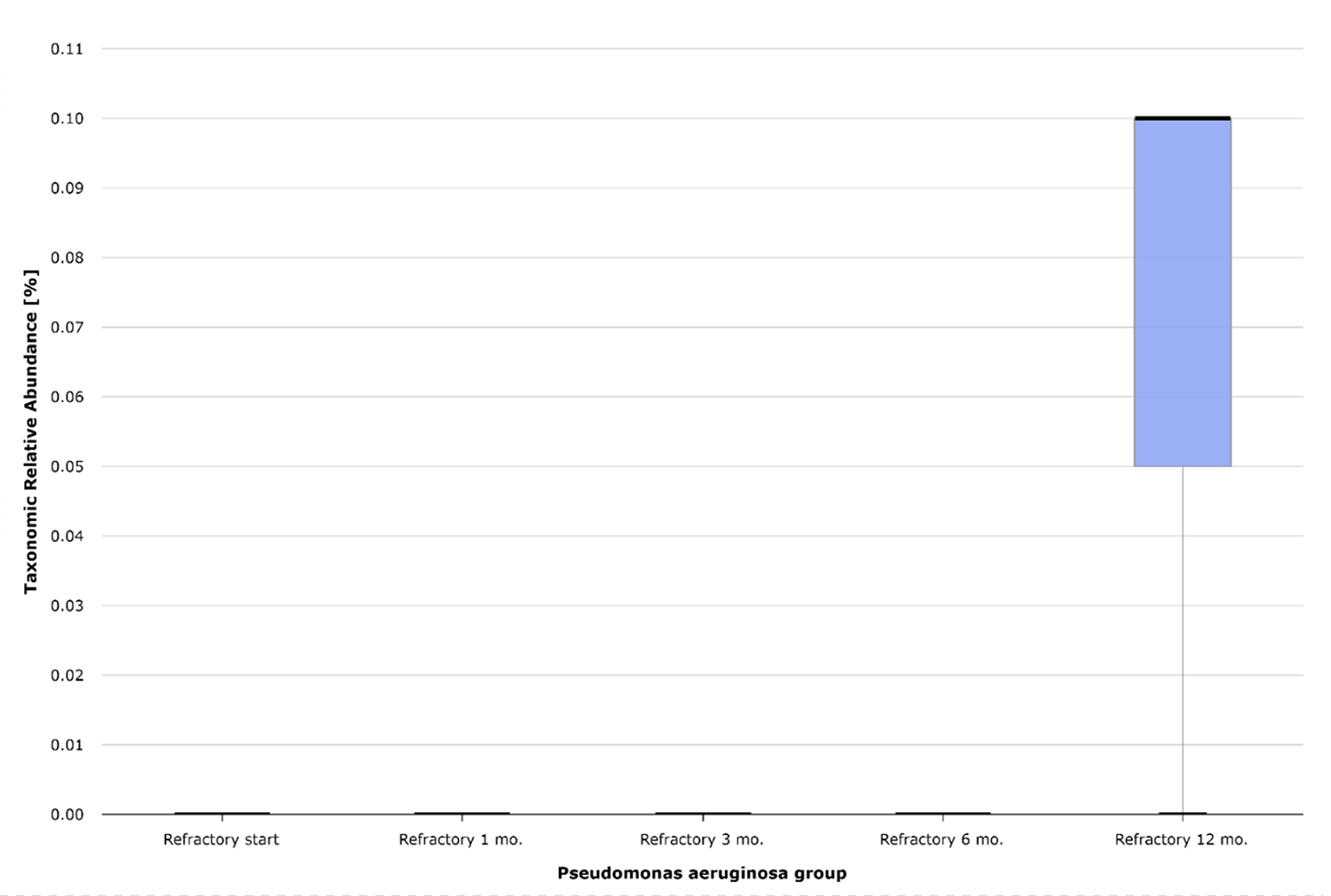

Supplement: Supplementary file 1 — Supplementary Information. [file 41598_2023_47230_MOESM1_ESM.docx]
